# A scoping review on information and support needs regarding cancer and work: part II—needs of employers and professionals in supporting cancer survivors

**DOI:** 10.1007/s11764-026-02019-z

**Published:** 2026-04-16

**Authors:** J. M. Mollet, M. A. Greidanus, S. F. A. Duijts, A. G. E. M. de Boer, P. M. V. Janse, I. L. Bakker, S. J. Tamminga

**Affiliations:** 1 Department of Public and Occupational Health, UMC Location University of Amsterdam, Amsterdam, The Netherlands; 2 Amsterdam Public Health Research Institute, Societal Participation and Health, Amsterdam, The Netherlands; 3 Netherlands Comprehensive Cancer Organization (IKNL), Research and Development, Utrecht, Netherlands; 4 Department of Medical Psychology, UMC Location Vrije Universiteit, Amsterdam, The Netherlands; 5 Cancer Center Amsterdam, Cancer Treatment and Quality of Life, Amsterdam, The Netherlands

**Keywords:** Cancer survivorship, Employment, Stakeholders, Scoping review, Information needs, Support needs

## Abstract

**Purpose:**

Employers and professionals can support cancer survivors during sick leave and return to work (RTW). However, they often face challenges in providing RTW support and existing interventions targeted at them are scarce and show limited effectiveness*.* This study aims to examine information and support needs of employers and professionals regarding sick leave and RTW of cancer survivors.

**Methods:**

For this scoping review, studies published between 2014 and 2024 were systematically searched across five databases. Included studies had to (1) involve employers (direct supervisors or HR managers) or professionals (healthcare and occupational health professionals); (2) use a qualitative, quantitative, or mixed method study design; and (3) report at least one information or support need in the results. Identified needs were qualitatively synthesized using the Arena in work disability prevention model.

**Results:**

Forty studies from 15 countries identified a diversity of needs across the personal, healthcare, workplace, and legislative systems. Employers and professionals need information and support on late effects of cancer and its treatment, providing RTW support, legislation, and multidisciplinary collaboration. Additionally, employers need information on communication, while professionals need support in assessing cancer survivors’ work capacity and functional abilities.

**Conclusions:**

Diverse information and support needs were identified. Some needs were universal, while others were context-dependent, as they depended on country-specific legislation. Multidisciplinary collaboration among stakeholders is essential, and contextual factors should be integrated into future interventions.

**Implications for cancer survivors:**

Addressing employers’ and professionals’ needs enables more comprehensive and personalized support for cancer survivors and strengthens RTW support systems.

**Supplementary Information:**

The online version contains supplementary material available at https://doi.org/10.1007/s11764-026-02019-z.

## Introduction

Multiple interventions have been developed over the years to enhance work participation of cancer survivors, as work has been shown to contribute to both quality of life [1, 2] and financial independence [3]. Many of such interventions have been carefully designed, often grounded in both theory and scientific evidence, and developed through stakeholder involvement [4–7]. However, most interventions focus exclusively on the cancer survivor, and despite all efforts, their effectiveness in supporting work participation remains limited in effectiveness studies.

The “Arena in work disability prevention model” (hereafter: Arena model) is a conceptual model that aims to describe the complexity of interacting systems in the case of work disability in a structured manner [8]. In the adapted version of the Arena model used in this study, the cancer survivor is presented to be surrounded by four main systems that influence work participation: the personal system (social relationships, affective, cognitive, and physical functioning), the healthcare system (variety of care management, such as multidisciplinary teams), the workplace system (work relatedness, employees assistance plans, workplace accommodation, including, for example, the organization and job position), and the legislative and insurance system (society’s safety net, such as provincial and federal legislation) [9]. Different stakeholders within each system can support cancer survivors during sick leave and return to work. Their active involvement is essential in designing interventions that facilitate work participation of cancer survivors. Employers, for example, are important stakeholders in the workplace system that can provide, among others, practical workplace accommodations and social support [10]. Both healthcare professionals and occupational health professionals are in the position to assess the work readiness of a cancer survivor, to provide recommendations regarding interventions, work schedules, or work adjustments [11], and to evaluate work disability for social security and/or insurance purposes. While healthcare professionals are clearly part of the healthcare system, occupational health professionals may operate across multiple systems, including the healthcare, workplace, and/or legislative or insurance system. This depends on a country’s legislation and the resulting specification of roles and responsibilities of stakeholders [12].

Stakeholders often experience challenges in providing effective support to cancer survivors during their sick leave and return to work and report a lack of information in providing this support. For example, employers may encounter difficulties in communication and decision-making [13], and healthcare professionals and occupational health professionals may experience a lack of knowledge about cancer or relevant legislation, faulty assumptions, or hesitancy regarding initiating work-related conversations [11, 14]. Despite growing awareness of (some of) these challenges, no comprehensive synthesis exists on the specific information and support employers and professionals need to effectively support cancer survivors. Recognizing and addressing the needs of employers and professionals is essential to enable them to provide comprehensive and personalized support to cancer survivors, thereby strengthening support systems across all phases of return to work ultimately facilitating holistic, tailored work-related support. This review adds value by systematically mapping the information and support needs of these key stakeholders across systems and professional roles. This approach not only highlights recurring gaps and barriers, but also identifies opportunities for targeted interventions, training, and policy alignment. This evidence base is essential for designing more effective, system-level strategies that go beyond the individual cancer survivor and address the broader ecosystem of return to work support.

Therefore, in this study, we aimed to systematically examine the information and support needs of employers and professionals regarding sick leave and return to work of cancer survivors.

## Methods

### Design

A scoping review was conducted as part of a broader research aim, i.e., to systematically examine the information and support needs of cancer survivors, employers, and professionals regarding sick leave and return to work of cancer survivors. The current article, Part II, focuses exclusively on the information and support needs of employers and professionals. The information and support needs of cancer survivors are described in Part I (ref.). The checklist Preferred Reporting Items for Systematic reviews and Meta-analyses extension for Scoping Reviews (PRISMA-ScR) was used as a framework to structure the reporting of this review [15]. The review protocol was not registered.

### Search strategy

One search strategy was developed to identify studies on the needs of cancer survivors, employers, and professionals. The search was conducted by an experienced clinical librarian and checked by two authors (ST and JM). The following databases were searched up to August 29, 2024: Medline, Embase, PsycINFO, Cinahl, and Scopus. Medical subject terms (MeSH terms) and title and abstract words related to “cancer,” “employment,” “worker,” “employer,” “professional,” “support,” “information,” and “needs” were combined and adapted for the different databases (Supplementary file A). The search strategy excluded randomized controlled trials by using the “NOT” operator. Filters were applied to limit all articles to those published after January 2014, written in English or Dutch, and available in full text. The publication timespan from 2014 or later was chosen because there has been substantial progress in cancer treatment and increased survival, which has led to evolving consequences for return to work of cancer survivors [16]. Therefore, we consider literature older than ten years to be no longer up to date.

### Article selection

Articles were included if they met the following criteria: (1) use of a quantitative (e.g., surveys), a qualitative (e.g., interviews and focus groups), or a mixed method (e.g., process evaluations or studies describing intervention development) study design; (2) an original peer-reviewed research article; (3) full text available in English or Dutch; (4a) for studies addressing the needs of *employers:* inclusion of a study sample of employers, including direct supervisors or human resource (HR) managers; (4b) for studies addressing the needs of *professionals:* inclusion of a study sample of professionals involved in care for workers diagnosed with cancer, including healthcare professionals (e.g., medical specialists) and occupational health professionals (e.g., occupational physicians); (5) description of at least one stakeholder’s information or support need regarding sick leave and return to work of cancer survivors in the results section. Studies were excluded if they employed an experimental design (e.g., randomized controlled trials), as such studies only report on needs as they are experienced in an experimental setting, which may differ from those in actual practice. Systematic reviews were also excluded to prevent data duplication and to ensure that only primary needs were captured.

The article selection process involved two steps. First, title and abstract screening was performed by two authors (ST and JM) simultaneously using the oracle mode in ASReview. The model was pre-trained (default setup: TF-IDF; Naïve Bayes; Maximum; Dynamic resampling) by uploading five relevant [13, 17–21] and five irrelevant research articles as prior knowledge. A stopping criterion of 50 consecutive irrelevant articles was used, assuming that all following articles would most likely not meet the inclusion criteria and should therefore be excluded (21). Second, a double-blind full-text screening was conducted by five authors (ST, MG, AdB, SD, and JM) using Covidence [22]. The articles that did not meet one or more of the inclusion criteria were excluded. In case of disagreement during the article selection, the articles were discussed between two authors (ST and JM) until consensus was reached.

### Data extraction and synthesis

Five authors performed data extraction (ST, MG, AdB, SD, and JM), with each paper being extracted by a single author. We used pre-designed data extraction tables capturing study characteristics (e.g., author, country, and design), study sample characteristics (e.g., number of participants and occupation), information needs, and support needs. First, needs were identified and extracted verbatim from the full texts and simultaneously classified as either an information or support need. Following this, the authors translated each identified need into a concise and summarized formulation. In case of doubt regarding the data extraction, discussions between three authors (ST, MG, and JM) were held until consensus was reached. Subsequently, the Arena model was used for the categorization of information and support needs. Within the main systems (i.e., personal, healthcare, workplace, and legislative and insurance system), subthemes were generated for each stakeholder (i.e., employer and professionals) by reviewing each identified need and clustering similar needs together by one author (JM) and checked and supplemented by another author (MG). For this data synthesis, we used the qualitative data analysis software MAXQDA [23]. Two stakeholder groups were distinguished: employers (or employers’ representative) and professionals. This distinction was made because healthcare and occupational health professionals are trained and qualified to provide support to cancer survivors, whereas employers typically provide support as part of their managerial responsibilities. To structure the identified needs, we applied definitions for both information and support needs. In the context of sick leave and return to work support, an information need refers to the need for relevant knowledge or resources required by employers and professionals to effectively support cancer survivors throughout the return to work process. A support need refers to any requirement or assistance necessary to strengthen the support provided to cancer survivors. These needs can cover multiple aspects of sick leave and return to work. This definition of information and support needs was inspired by previous needs-related research conducted in a different population [24].

## Results

### Characteristics of included studies

**Fig. 1:**
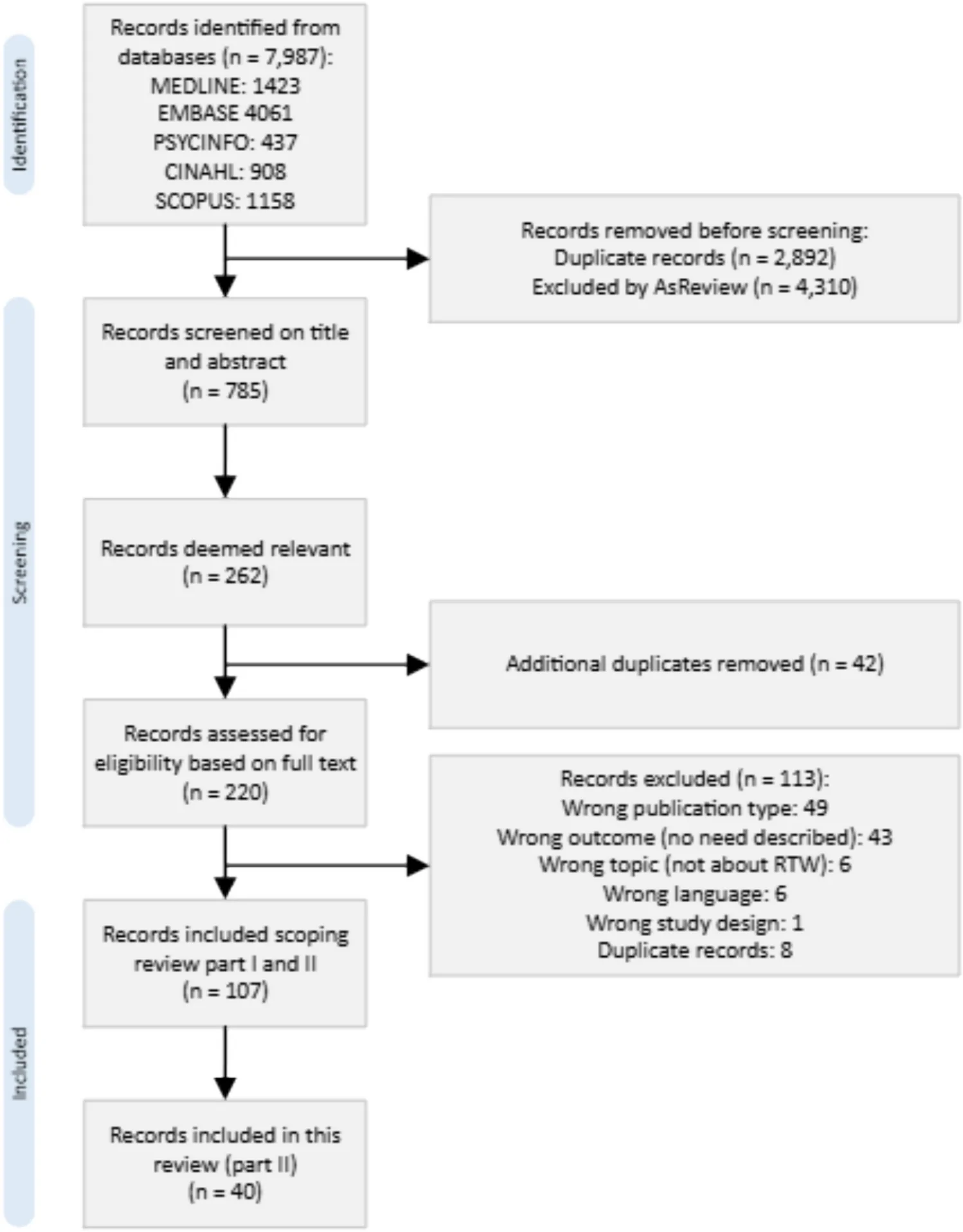
PRISMA flowchart of study selection and screening

In total, our search strategy identified 7987 records, of which 2892 were duplicates. Using the stopping criterion of 50 consecutive irrelevant records, a total of 785 records were screened on title and abstract, of which 262 met the inclusion criteria. After removing 42 duplicates, a total of 220 full texts were screened, of which 107 articles met our inclusion criteria and were therefore included in either Part I (85 articles) or Part II, or both if both stakeholder groups were included (18 articles). Duplicates were identified at several stages, as the same record appearing in different databases is not always automatically detected as a duplicate. Of these 107 articles, 40 specifically addressed the aim of the current study—Part II—concerning the information and support needs of employers and professionals (Fig. 1) and were therefore included in this study.

The 40 included articles were conducted in 14 different countries, with the majority conducted in The Netherlands (*N* = 17, 42.5%), Canada, and Belgium (both *N* = 4, 10%), followed by Australia and France (each *N* = 3, 7.5%). Thirty-eight articles were cross-sectional, and two articles were longitudinal. Eleven articles identified the needs of employers, 22 articles identified the needs of professionals, and 7 articles described both employers’ and professionals’ needs. Some needs initially appeared to belong to more than one Arena model system, as the system in which a need was perceived differed from the system expected to provide or address that need. This was handled by assigning needs based on the definitions of the Arena model systems, independent of the source expected to fulfill or address the need. Employers were represented by several occupations, e.g., department managers and HR managers, while also a wide range of professionals were represented, e.g., oncologists, occupational physicians, nurses, psychologists, and physiotherapists. An overview of all study characteristics is presented in Table 1. More detailed information on the extracted occupations (of professionals) and needs can be found in supplementary file B.

**Table 1:** Study characteristics

Study characteristics (*N* = 40)	*N* (%)
*Countries*	
Netherlands	17 (43%)
Canada	4 (10%)
Belgium	4 (10%)
Australia	3 (8%)
France	3 (8%)
Other	9 (23%)
*Study design*	
Cross-sectional	38 (98%)
Longitudinal	2 (5%)
*Methodology*	
Questionnaire	8 (20%)
Interview	33 (83%)
Focus group	11 (28%)
*Stakeholder*	
Employer	18 (45%)
Professional	29 (73%)

The total number may be higher as a result of incorporating different methodologies

### Information and support needs

**Fig. 2:**
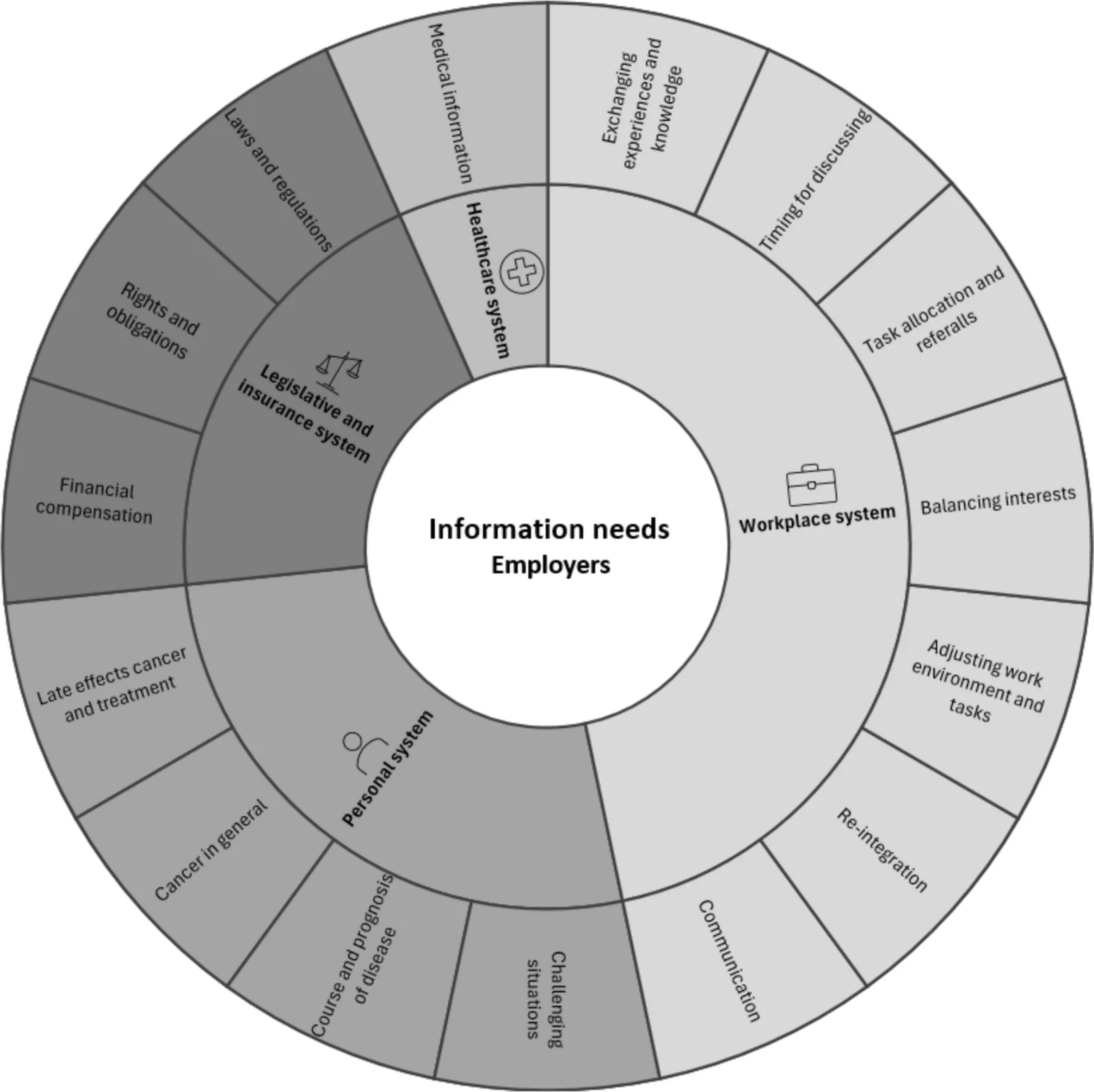
Identified themes of employers’ information needs corresponding to each of the four systems of the Arena model

**Fig. 3:**
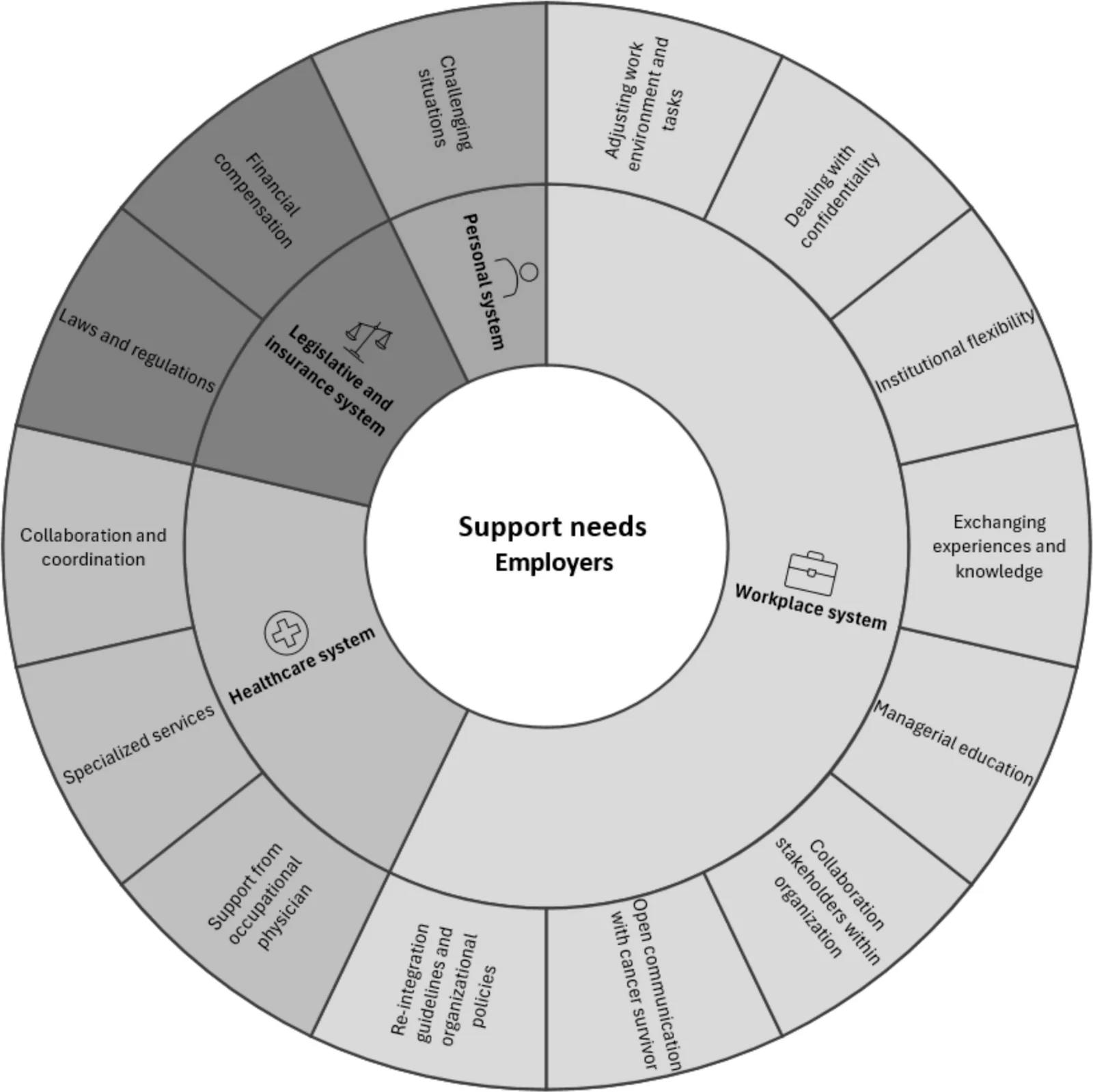
Identified themes of employers’ support needs corresponding to each of the four systems of the Arena model

**Fig. 4:**
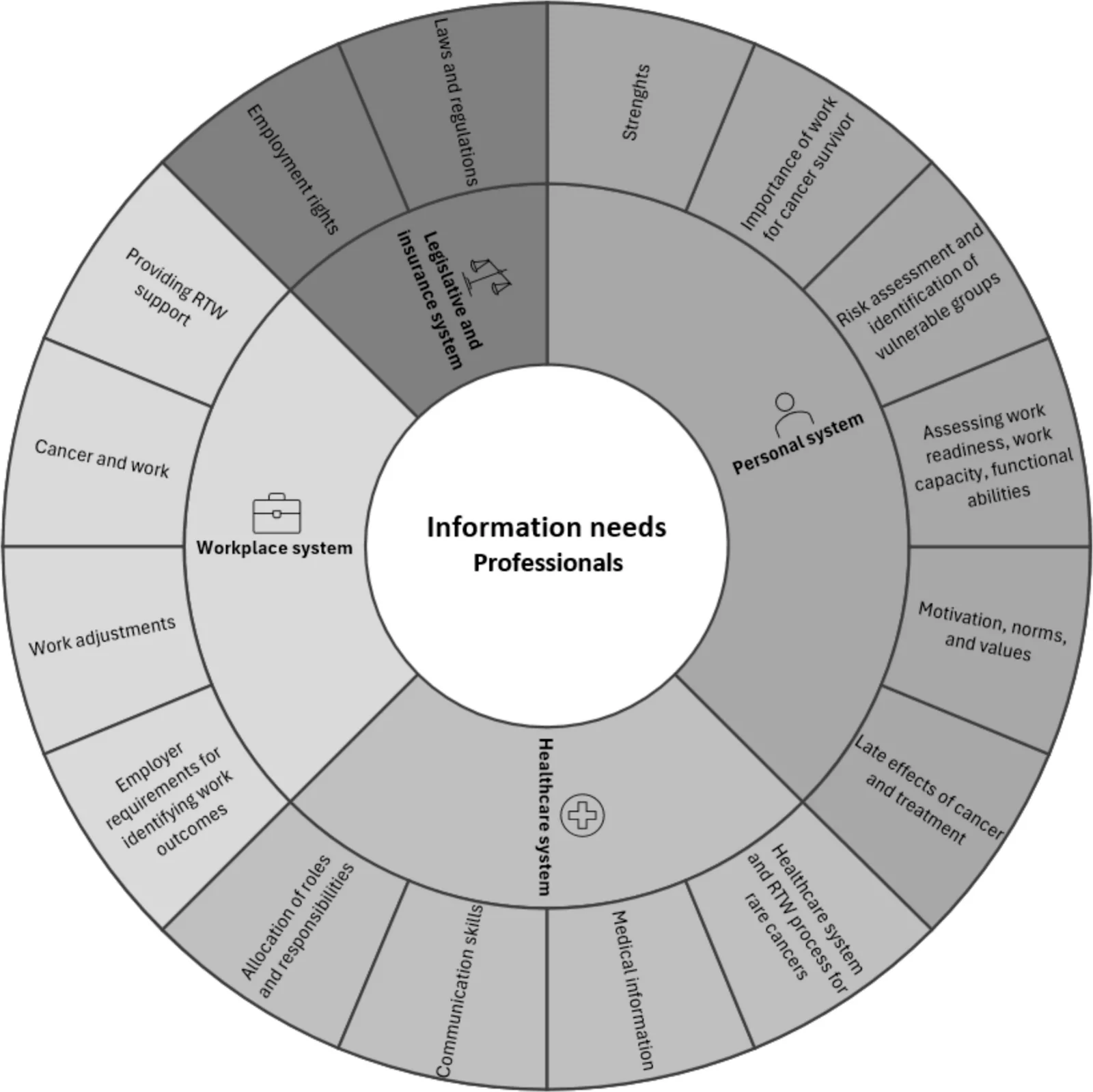
Identified themes of professionals’ information needs corresponding to each of the four systems of the Arena model

**Fig. 5:**
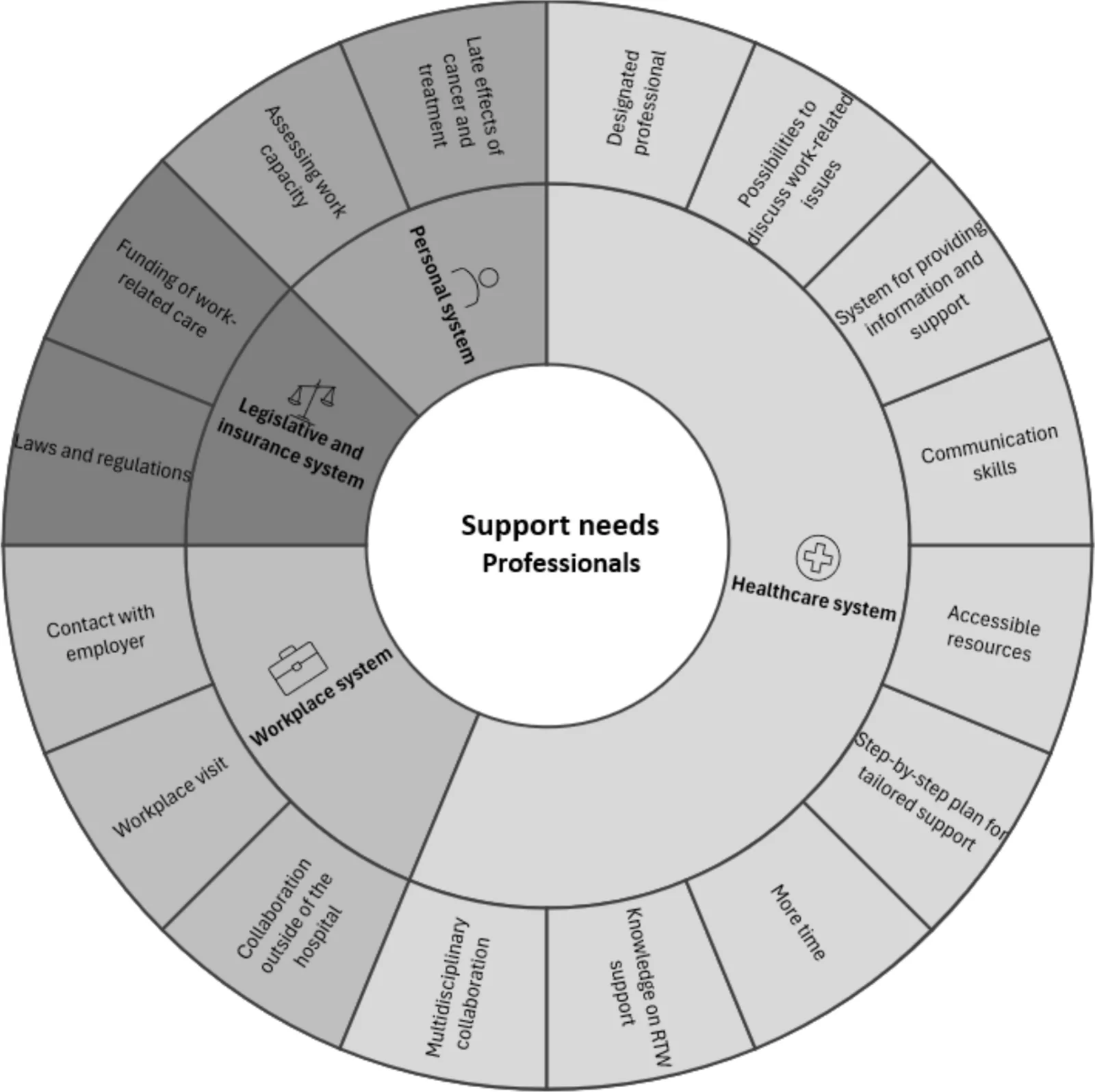
Identified themes of professionals’ support needs corresponding to each of the four systems of the Arena model

A synthesis of the information and support needs of employers and professionals is presented consecutively, organized by system according to the Arena model. All identified information and support needs of employers are summarized in Figs. 2 and 3, and those of professionals in Figs. 4 and 5. Certain identified needs were universal, while others were context-dependent due to differences in national legislation.

#### Employers

##### Personal system

Employers in several studies expressed the need for information about *cancer in general *[25–27] –e.g., on the *course and prognosis of the disease* [27–29] – and on *late effects of cancer and its treatment* in relation to work ability [26, 29–31]. Employers also reported multiple *challenging situations* in which they felt a need for information and support [13, 25, 32], i.e., when supporting cancer survivors who are unable to return to work [25], terminally ill [13, 25, 32], experiencing emotional distress [25], unwilling to participate in their reintegration process [25], or experiencing long-term sick leave [32].

##### Workplace system

Several studies have identified a need among employers for information on *re-integration* in general and in particular supporting cancer survivors during their return to work [28, 33-36]. Employers also mentioned that they would appreciate receiving specific information and support regarding *adjusting work environment and work tasks* (e.g., from an occupational physician) [13, 28, 37, 38], *balancing the interests* of the employee with cancer with the interest of the organization [13, 36], and *dealing with confidentiality* [33]. Across multiple studies, employers expressed a support need for structured *reintegration guidelines and organizational policies* to help navigate the return to work trajectory of cancer survivors [27]. On the other hand, they emphasized a support need for *institutional flexibility* to provide tailored support and to address specific situations effectively [25, 39]. Employers also identified a need for *managerial education* regarding supporting cancer survivors [33, 39].

Employers in various studies mentioned the need for information and training about *communication* [13, 27, 29, 38, 41], e.g., how to keep in contact with the cancer survivor, how to keep colleagues informed while respecting the privacy of the cancer survivor, and how to communicate in case of an unfavorable prognosis or in the event of death. Moreover, employers were found to experience the need for *open communication with the cancer survivor* in order to identify individual needs of the cancer survivor [29] and to ensure that services are available in time [39]. In addition, employers mentioned a need for information about the *timing for discussing* important work-related matters, such as work ability and adjusting work environment and work tasks [13].

Employers indicated a need for support in fostering *collaboration between all involved stakeholders within the organization*, such as HR managers, administrators, and the direct supervisor, particularly regarding work-related matters and available leave entitlements [39]. Additionally, they need information on *task allocation and referrals* to healthcare providers [25, 38] and where to access specialized support [38]. A further information and support need concerns the *exchange of experiences and knowledge* with physicians or fellow employers, for instance through a forum [25, 27].

##### Healthcare system

In multiple studies, employers’ support need for *collaboration and coordination* with healthcare professionals and occupational health professionals [29, 33, 35, 42] was emphasized. In one study, employers specifically noted the need for *support from an occupational physician* or other external experts, both to assist the cancer survivor and themselves in managing the return to work trajectory [28]. To facilitate adequate work accommodations, they also require access to specific *medical information* from those professionals [43]. In complex cases, employers mentioned a need for support from *specialized services*, such as specialized re-integration services [25], and information on where to access such resources [25, 38].

##### Legislation and insurance system

In various studies, employers indicated a need for information and support regarding *laws and regulations* concerning return to work in general [25, 27, 41]. One study specifically highlighted the need for information on legislation that adapts to different situations and that also guarantees job retention after sick leave [35], and another study emphasized the need for supportive vocational rehabilitation policies [42]. Employers also seek to enhance their knowledge regarding their *rights and obligations* [41] and information on possibilities for *financial compensation* that incentivizes to keep the sick-listed cancer survivor employed [28, 40] and to support cancer survivors [29].

#### Professionals

##### Personal system

In multiple studies, professionals expressed a need for information about *late effects of cancer and treatment*, particularly in relation to work capacity [14, 26, 44–46]. They emphasized the importance of understanding barriers and medically related limitations or restrictions that hinder return to work for cancer survivors. A specific support need related to late effects of cancer and treatment was mentioned for screening programs targeting long-term cancer-related effects, such as cognitive problems [47] and for tools to objectively *assessing the work readiness, work capacity, and functional abilities* of cancer survivors [46, 48].

In other studies, professionals’ information needs for insights into *motivation, norms, and values* that cancer survivors hold [33, 50] were highlighted and what *strengths* a cancer survivor is bringing to their work [46]. Also, professionals mentioned a need for knowledge on the individual *importance of work for the cancer survivor* [51].

Finally, professionals emphasized the information need for guidelines and criteria to support *risk assessment and the identification of vulnerable groups*, for example, on risk assessment of long-term sick leave or disability after cancer [33], or a set of standard criteria to identify socially deprived cancer survivors [33].

##### Workplace system

Professionals need information about *cancer*—both in general and specifically regarding rare cancers—*and work *[18, 45, 52]. In various studies, professionals indicated a need for specific information on *providing return to work support* to cancer survivors [33, 38, 52], including information on timing for discussing return to work [51] and available return to work interventions and resources [38, 51, 52]. Occupational physicians indicated a specific information need for training which includes among other aspects guidelines for *work adjustments* [48].

Both healthcare professionals and occupational health professionals highlighted the support need for *collaboration with professionals outside the hospital* [53] and with the workplace environment [5]. They emphasized the importance of staying in *contact with the employer* [5]. In addition, occupational therapists described the need for *workplace visits* in order to get a clear understanding of the occupation and activities of the cancer survivor [50]. Physicians in another study expressed an information need on *the employer’s requirements for identifying work outcomes* after cancer [45].

##### Healthcare system

Several studies highlighted professionals’ support need for *multidisciplinary collaboration* [33, 43, 44, 46, 49, 50, 54-57]. Also, a need for information was expressed on clear *allocation of roles and responsibilities* among key stakeholders involved in return to work trajectories [45, 53, 56, 58] and referring cancer survivors to other professionals [38, 59]. This mainly concerns collaboration between healthcare professionals and occupational health professionals.

Occupational physicians expressed a need for medical information, because they often lack access to information on treatment and the clinical course, due to not working in the clinical setting [35]. General practitioners similarly indicated the information and support need for clear communication with oncologists, particularly regarding the end of treatment and the appropriate timing for initiating aftercare [56]. Professionals also mentioned the support need for possibilities to discuss work-related issues of cancer survivors during multidisciplinary meetings, as these topics are often not part of these meetings’ agenda [51]. 

Occupational health professionals expressed the support need for *more time* for the first consultation with a cancer survivor [60] and to address work-related issues, such as return-to-work procedures and legal issues [51]. Moreover, professionals mentioned the support need for a *step-by-step plan to provide cancer survivors with tailored support* and the need for a *designated professional* who is responsible for addressing all work-related issues faced by the cancer survivor [53].

Professionals reported the need for *knowledge on return to work support *[38, 51, 60, 61], including training in how to address the work-related challenges cancer survivors might encounter [51] or a refresher course [60]. Additionally, professionals expressed the need for a better *system to provide work-related information and support* to cancer survivors [38] and *accessible resources* to support all cancer survivors during their return to work [52], along with training to increase awareness of those available support resources [38]. Additionally, professionals indicated a need for training to enhance their *communication skills* [38]. More specifically, studies including occupational physicians and vocational rehabilitation consultants highlighted the importance of targeted training and information to support communication around sensitive topics, such as prognosis and functional outcomes [45, 62]. In a study on rare cancers, professionals expressed a need for information about the *healthcare system and return to work process for patients with a rare cancer *[18]. Rare cancer survivors often experience having to navigate the system on their own, due to limited general and return to work-related information concerning rare cancers, compared to common cancers.

##### Legislative and insurance system

Finally, several studies reported that professionals expressed a need for information and support on laws and regulations regarding sick leave, disability, return to work [14, 53, 63], and information on *employment rights*, specifically after cancer [30]. Professionals also highlighted the need for *funding to support the provision of work-related care* [54, 64]. Financial support from health insurers to conduct in-depth evaluations of functional abilities of cancer survivors is also a need mentioned by professionals [46].

## Discussion

### Main findings and interpretation

In this study, we aimed to systematically examine the information and support needs of employers and professionals regarding sick leave and return to work of cancer survivors. A total of 40 studies were included, identifying a large variety of needs, which varied both between and within stakeholders. Specific needs identified for both employers and professionals include a need for information on the late effects of cancer and cancer treatment specifically in relation to work capacity, how to support cancer survivors during their return to work, and legislation regarding return to work. Additionally, this review indicated the profound need for collaboration between all stakeholders within the healthcare system and the workplace system. Specific needs for employers included the need for information about how to communicate with the cancer survivor. Professional-specific needs entailed support in objectively assessing the work capacity and functional abilities of cancer survivors.

While some needs are universal, such as the need for collaboration, the information and support needs identified in this review vary significantly both between and within stakeholders. This may be explained by differences in the sociopolitical context in which the included studies were conducted. Broader institutional and economic contexts shape (the distribution of) responsibilities in the sick leave and return to work processes and thus also influence the information and support needs experienced by professionals and employers in these different contexts. For example, cross-country differences exist in the following: (1) sickness insurance schemes [12], (2) the extent to which work is integrated into curative care [65], and (3) legislation regarding and access to occupational health and related services [66]. Studies included in this review predominantly originate from the Netherlands, a country where legislation dictates that the employer bears significant responsibility both in terms of reintegration and continued salary payments [67]. Moreover, the involvement of an occupational physician is mandatory in the Dutch occupational healthcare system [67]. Within this context, employers in the Netherlands expressed needs for financial compensation to incentivize retention of sick listed employees and for support from occupational physicians and specialized services, while professionals highlighted a need for more time to discuss work participation. This is, for example, contrasted by a country like Romania, where a coordinated approach is lacking and institutional support for the return to work of sick-listed employees is limited [68]. Therefore, a need identified in this review among Romanian employers is the establishment of legislation to support employees after returning to work, particularly legislation regarding vocational rehabilitation. As some stakeholders’ information and support needs are thus context-dependent, national and organizational policies should be considered when determining which stakeholder needs are relevant and how they should be addressed. The findings of this review could well serve as a foundation for developing interventions in different countries. The needs can serve as a starting point for conducting a thorough needs assessment to guide intervention development, for example, within a structured intervention mapping (IM) approach [69]. As such, interventions for professionals and employers can be aligned with the needs they experience in the specific context in which they operate.

Comparing the identified needs of stakeholders in this review (Part II) with those of cancer survivors (Part I) [70] revealed several similarities, such as the need for information, communication, and collaboration, as well as some discrepancies. It may be relevant to compare the needs of cancer survivors with those of professionals and employers. However, a need expressed by one stakeholder may be addressed by another stakeholder, which does not necessarily reflect the latter’s own needs. For example, both cancer survivors and employers reported a need for information about cancer and the late effects of diagnosis and treatment. Professionals would be the most appropriate group to provide this information. However, also professionals themselves expressed a need for more knowledge on this topic. In addition, both professionals and employers indicated a need for more medical information. Providing such information, however, is not always possible or permitted, and cancer survivors emphasized the importance of maintaining their privacy. Moreover, employers expressed a need for information on timing for discussing return to work, which aligns with cancer survivors’ need for information about the timing of work resumption. For cancer survivors, these needs extend even further. For instance, they require more information about the different phases of return to work and sick leave, and they expressed a need for both early and long-term support throughout this process. At last, we found in this review that employers and professionals indicated a need for collaboration between involved stakeholders. This aligns with previously identified patients’ needs for more collaboration between their health professional and occupational health professional [70, 71]. Such collaboration is important to jointly address patients’ work-related concerns and to provide insights into the consequences of their diagnosis and its treatment on patients’ ability to work. Moreover, working in multidisciplinary teams facilitates referral to other relevant stakeholders and enables a more holistic approach [71]. Previous research has also indicated that the multidisciplinary nature of interventions could be crucial for enhancing work participation of cancer survivors [72]. However, coordination between stakeholders is often lacking within existing systems, which can be a barrier for return to work [65]. Enhancing interdisciplinary communication and collaboration thus needs to be addressed in future interventions.

### Strengths and limitations

A key strength of this scoping review is that it addresses a gap in the existing knowledge about the stakeholders surrounding cancer survivors, whereas most research has focused primarily on the survivors themselves. Moreover, this comprehensive overview of the information and support needs of employers and professionals is directly applicable as a starting point for the development of new interventions aimed at these stakeholders. However, there are also certain limitations which need to be addressed. Nearly half of the included studies were conducted in the Netherlands, a country where there has apparently been considerable research attention to the topic of cancer and work, while studies from regions such as Africa and South America were notably absent. As some stakeholders’ information and support needs are shaped by sociopolitical and economic contexts, the findings may therefore not be fully generalizable to the underrepresented countries. Lastly, throughout the review, certain aspects proved difficult to interpret: (1) whether stakeholders were expressing a ‘need’ or merely describing an “experience” and (2) whether to differentiate between a need for information or a need for support. To address these ambiguities, several authors have repeatedly discussed borderline cases together to reach shared and consistent interpretations. The raw data extracted from the included studies have been provided in the supplementary file to ensure transparency.

### Implications for clinical practice and future research

The needs identified in this review can serve as a foundation in the development of interventions targeting employers and professionals. So far, evidence- and theory-based interventions specifically aimed at these stakeholders remain limited. The results of this review highlight the importance of prioritizing collaboration between stakeholders involved in the cancer survivors’ sick leave and return to work, both in research and clinical practice. For example, lowering the threshold to engage with other stakeholders and improving communication between them can help strengthen collaboration [73]. Therefore, we recommend the development of interventions that offer a system approach, focusing on enhancing communication and collaboration among employers and professionals, while also integrating the needs of cancer survivors identified in Part I [70]. For example, it is recommended to elaborate on the work-oriented care model that is developed in previous research [74], which incorporated collaboration between healthcare professionals and an occupational physician [74]. Moreover, to further enhance the applicability of the Arena model, we propose the addition of a component that explicitly addresses collaboration between the independent systems. This would allow future research and interventions to target not only individual systems, but also the coordination and collaboration between them. We also recommend future research to focus on identifying where and/or from whom employers and professionals wish to receive this information and support, while also considering the availability of resources within specific countries and contexts, as this is currently lacking in the literature. Lastly, we recommend exploring stakeholder needs across different national contexts. A country-level analysis would be valuable to better align future interventions with the specific information and support needs of stakeholders within each country.

## Conclusion

A large variation in information and support needs was identified among employers and professionals involved in the cancer survivor’ sick leave and return to work. These needs are often shaped by sociopolitical and economic contexts. There is also a strong need for improved collaboration among stakeholders, for instance, through improved communication and lowering the threshold to engage with other stakeholders. Future interventions should therefore be multidisciplinary and tailored to the sociopolitical and economic context.

## Supplementary Information

Below is the link to the electronic supplementary material.

ESM 1 (DOCX  37.5 KB)

ESM 2 (DOCX 97.8 KB)

## Data Availability

The datasets are available in the supplementary files.
