# Supplementary material for: A scoping review on information and support needs regarding cancer and work: part II—needs of employers and professionals in supporting cancer survivors
Source: J Cancer Surviv. 2026 Apr 16;20(Suppl 2):2472–85. doi: 10.1007/s11764-026-02019-z (PMC13744526; doi:10.1007/s11764-026-02019-z)
Supplement: Supplementary file 1 [file 11764_2026_2019_MOESM1_ESM.docx]

**Supplementary file A – search**

**2-9-2024:**

| Databases: | Before deduplication | After deduplication* |
| --- | --- | --- |
| Medline (Ovid)  Embase (Ovid)  Psycinfo (Ovid)  Cinahl (Ebsco)  Scopus (Elsevier) |  |  |
| Total | 7987 | 5095 |

*Lobbestael, G. (2023). DedupEndNote (Version 1.0.0) [Computer software]. https://github.com/globbestael/DedupEndNote

MEDLINE (OVID):

Database(s): Ovid MEDLINE(R) Epub Ahead of Print and In-Process, In-Data-Review & Other Non-Indexed Citations and Daily August 29, 2024
Search Strategy:

| # | Searches | Results |
| --- | --- | --- |
| 1 | exp Neoplasms/ or Cancer Survivors/ | 4012773 |
| 2 | (cancer* or neoplas* or carcino* or adenocarino* or oncolog* or malignan* or tumo* or leukemi* or sarcoma* or lymphom* or melanom* or blastoma*).ti,ab,kf. | 4680094 |
| 3 | 1 or 2 | 5501845 |
| 4 | exp Return to Work/ or exp Sickness Impact Profile/ or exp Absenteeism/ or exp Convalescence/ or exp Sick Leave/ or exp Work Capacity Evaluation/ or exp Rehabilitation, Vocational/ | 45040 |
| 5 | ("return to work" or rtw).ti,ab,kf. | 12433 |
| 6 | ((work or worker* or employee* or occupational*) adj3 performanc*).ti,ab,kf. | 12982 |
| 7 | ((work or worker* or occupation* or employee*) adj3 (absen* or back or reintegration* or capacity or disability or incapacity or incapability or retention or rehabilitation or recovery or productivity or participation or workability or "medical leave" or "sick leave" or "disability leave" or "disability absence" or convalescen* or sick day* or sick listed* or sicklisted or ilness day* or reemployment or "job reentry" or presenteeism or "sickness absence" or "work day loss" or "work time loss")).ti,ab,kf. | 35742 |
| 8 | 4 or 5 or 6 or 7 | 90514 |
| 9 | "health services needs and demand"/ or needs assessment/ or Information Services/ or exp Information Sources/ or "Attitude of Health Personnel"/ or employment, supported/ or exp workplace/ or help-seeking behavior/ or "Patient Education as Topic"/ or Health Knowledge, Attitudes, Practice/ or "Standard of Care"/ or exp social support/ or psychosocial support systems/ or Self-Help Groups/ or Occupational Health/st | 1874340 |
| 10 | (need* or experienc* or tool* or program* or care standard* or guideline* or challeng* or prefer* or wish* or obstacle* or worry or worries or confidence or empower* or self-management or education or perception* or demand or health literacy).ti,ab,kf. | 8144683 |
| 11 | (support* adj3 (occupation* or work* or employ* or professional* or social or information* or survivor* or strateg*)).ti,ab,kf. | 140168 |
| 12 | 9 or 10 or 11 | 9458603 |
| 13 | 3 and 8 and 12 | 2926 |
| 14 | limit 13 to yr="2014 -Current" | 1576 |
| 15 | exp Randomized Controlled Trial/ or Controlled Clinical Trial/ or "Randomized Controlled Trials as Topic"/ or Random Allocation/ or Single-Blind Method/ or Double-Blind Method/ | 990138 |
| 16 | (random* adj3 trial*).ti,ab,kf. | 521961 |
| 17 | (randomi?ation or random allocation or rct).ti,ab,kf. | 98135 |
| 18 | 15 or 16 or 17 | 1229172 |
| 19 | 14 not 18 | 1423 |

EMBASE (OVID):

Database(s): Embase Classic+Embase 1947 to 2024 August 30
Search Strategy:

| # | Searches | Results |
| --- | --- | --- |
| 1 | exp neoplasm/ or cancer survivor/ | 6306107 |
| 2 | (cancer* or neoplas* or carcino* or adenocarino* or oncolog* or malignan* or tumo* or leukemi* or sarcoma* or lymphom* or melanom* or blastoma*).ti,ab,kf. | 6603597 |
| 3 | 1 or 2 | 7854397 |
| 4 | return to work/ or sickness impact profile/ or absenteeism/ or convalescence/ or medical leave/ or work capacity/ or vocational rehabilitation/ | 131500 |
| 5 | ("return to work" or rtw).ti,ab,kf. | 16882 |
| 6 | ((work or worker* or employee* or occupational*) adj3 performanc*).ti,ab,kf. | 15981 |
| 7 | ((work or worker* or occupation* or employee*) adj3 (absen* or back or reintegration* or capacity or disability or incapacity or incapability or retention or rehabilitation or recovery or productivity or participation or workability or "medical leave" or "sick leave" or "disability leave" or "disability absence" or convalescen* or sick day* or sick listed* or sicklisted or ilness day* or reemployment or "job reentry" or presenteeism or "sickness absence" or "work day loss" or "work time loss")).ti,ab,kf. | 51079 |
| 8 | 4 or 5 or 6 or 7 | 185844 |
| 9 | needs assessment/ or personal needs/ or personal experience/ or supported employment/ or workplace/ or information service/ or information seeking/ or information source/ or patient education/ or help seeking behavior/ or exp health literacy/ or exp health personnel attitude/ or attitude to health/ | 653224 |
| 10 | (need* or experienc* or tool* or program* or care standard* or guideline* or challeng* or prefer* or wish* or obstacle* or worry or worries or confidence or empower* or self-management or education or perception* or demand or health literacy).ti,ab,kf. | 10922417 |
| 11 | (support* adj3 (occupation* or work* or employ* or professional* or social or information* or survivor* or strateg*)).ti,ab,kf. | 185685 |
| 12 | 9 or 10 or 11 | 11228034 |
| 13 | 3 and 8 and 12 | 7331 |
| 14 | limit 13 to yr="2014 -Current" | 4555 |
| 15 | exp randomized controlled trial/ or controlled clinical trial/ or "randomized controlled trial (topic)"/ or exp randomization/ or single blind procedure/ or double blind procedure/ | 1432046 |
| 16 | (random* adj3 trial*).ti,ab,kf. | 709188 |
| 17 | (randomi?ation or random allocation or rct).ti,ab,kf. | 153835 |
| 18 | 15 or 16 or 17 | 1675912 |
| 19 | 14 not 18 | 4061 |

PSYCINFO (OVID):

Database(s): APA PsycInfo 1806 to August 2024 Week 5
Search Strategy:

| # | Searches | Results |
| --- | --- | --- |
| 1 | exp neoplasms/ | 65237 |
| 2 | (cancer* or neoplas* or carcino* or adenocarino* or oncolog* or malignan* or tumo* or leukemi* or sarcoma* or lymphom* or melanom* or blastoma*).ti,ab,id. | 103591 |
| 3 | 1 or 2 | 105262 |
| 4 | reemployment/ or employee absenteeism/ or exp employment status/ or vocational rehabilitation/ | 40907 |
| 5 | ("return to work" or rtw).ti,ab,id. | 3408 |
| 6 | ((work or worker* or employee* or occupational*) adj3 performanc*).ti,ab,id. | 13935 |
| 7 | ((work or worker or workers or occupation* or employee*) adj3 (absen* or back or reintegration* or capacity or disability or incapacity or incapability or retention or rehabilitation or recovery or productivity or participation or workability or "medical leave" or "sick leave" or "disability leave" or "disability absence" or convalescen* or sick day* or sick listed* or sicklisted or ilness day* or reemployment or "job reentry" or presenteeism or "sickness absence" or "work day loss" or "work time loss")).ti,ab,id. | 19746 |
| 8 | 4 or 5 or 6 or 7 | 70057 |
| 9 | needs assessment/ or health service needs/ or exp needs/ or psychological needs/ or special needs/ or work adjustment training/ or supported employment/ or exp employee benefits/ or employee assistance programs/ or vocational evaluation/ or rehabilitation counseling/ or exp Health Literacy/ | 45781 |
| 10 | (need* or experienc* or tool* or program* or care standard* or guideline* or challeng* or prefer* or wish* or obstacle* or worry or worries or confidence or empower* or self-management or education or perception* or demand or health literacy).ti,ab,id. | 2634503 |
| 11 | 9 or 10 | 2638649 |
| 12 | 3 and 8 and 11 | 667 |
| 13 | limit 12 to yr="2014 -Current" | 450 |
| 14 | Randomized Controlled Trials/ | 1099 |
| 15 | (random* adj3 trial*).ti,ab,id. | 75535 |
| 16 | (randomi?ation or random allocation or rct).ti,ab,id. | 15051 |
| 17 | 14 or 15 or 16 | 81939 |
| 18 | 13 not 17 | 437 |

CINAHL (EBSCO):

908 hits:

Publication Date: 20140101-20240831

( (MH "Neoplasms+") OR (MH "Cancer Survivors") ) OR TI ( cancer* or neoplas* or carcino* or adenocarino* or oncolog* or malignan* or tumo* or leukemi* or sarcoma* or lymphom* or melanom* or blastoma* ) OR AB ( cancer* or neoplas* or carcino* or adenocarino* or oncolog* or malignan* or tumo* or leukemi* or sarcoma* or lymphom* or melanom* or blastoma* )

AND

( (MH "Job Re-Entry") OR (MH "Absenteeism") OR (MH "Presenteeism") OR (MH "Impairment, Health Professional") OR (MH "Sick Leave") OR (MH "Work Capacity Evaluation") OR (MH "Rehabilitation, Vocational") OR (MH "Sickness Impact Profile") ) OR TI ( "return to work" or rtw ) OR AB ( "return to work" or rtw ) OR TI ( (work* or employee* or occupational*) N1 performanc* ) OR AB ( (work* or employee* or occupational*) N1 performanc* ) OR TI ( (work or worker or workers or occupation* or employee*) N3 (absen* or back or reintegration* or capacity or disability or incapacity or incapability or retention or rehabilitation or recovery or productivity or participation or workability or "medical leave" or "sick leave" or "disability leave" or "disability absence" or convalescen* or sick day* or sick listed* or sicklisted or ilness day* or reemployment or "job reentry" or presenteeism or "sickness absence" or "work day loss" or "work time loss") ) OR AB ( (work or worker or workers or occupation* or employee*) N3 (absen* or back or reintegration* or capacity or disability or incapacity or incapability or retention or rehabilitation or recovery or productivity or participation or workability or "medical leave" or "sick leave" or "disability leave" or "disability absence" or convalescen* or sick day* or sick listed* or sicklisted or ilness day* or reemployment or "job reentry" or presenteeism or "sickness absence" or "work day loss" or "work time loss") )

AND

( (MH "Needs Assessment") OR (MH "Information Needs") OR (MH "Human Needs (Psychology)+") OR ( (MH "Health Services Needs and Demand+") ) OR (MH "Information Resources+") OR (MH "Health Information+") OR (MH "Support, Psychosocial") OR (MH "Employment, Supported") OR (MH "Patient Education") OR (MH "Health Literacy")) OR ( TI (need* or experienc* or tool* or program* or care standard* or guideline* or challeng* or prefer* or wish* or obstacle* or worry or worries or confidence or empower* or self-management or education or perception* or demand or health literacy) OR AB (need* or experienc* or tool* or program* or care standard* or guideline* or challeng* or prefer* or wish* or obstacle* or worry or worries or confidence or empower* or self-management or education or perception* or demand or health literacy) ) OR ( TI ( support* N3 (occupation* or work* or employ* or professional* or social or information* or survivor* ) OR AB ( support* N3 (occupation* or work* or employ* or professional* or social or information* or survivor* ) )

NOT

( (MH "Randomized Controlled Trials+") OR (MH "Double-Blind Studies") OR (MH "Single-Blind Studies") ) OR (MH "Random Assignment") OR ( TI ( randomization or randomisation or random allocation or rct ) OR AB ( randomization or randomisation or random allocation or rct ) OR TI random* N3 trial* OR AB random* N3 trial* )

SCOPUS (Elsevier)

1158 results:

( ( TITLE-ABS-KEY ( cancer* OR neoplas* OR carcino* OR adenocarino* OR oncolog* ) AND TITLE-ABS-KEY ( "return to work" OR rtw OR "sick leave" OR reemployment OR "job reentry" OR "sickness absence" OR "vocational rehabilitation" OR reintegration* ) AND TITLE-ABS-KEY ( need* OR experienc* OR tool* OR program* OR "care standard*" OR guideline* OR education OR perception* OR preference* OR demand OR "supported employment" OR support OR "health attitude*" OR "health information*" OR "information source*" OR "information service*" OR "health literacy" ) ) AND PUBYEAR > 2013 AND PUBYEAR < 2025 ) AND NOT ( TITLE-ABS-KEY ( "randomized controlled trial*" OR randomization OR randomisation OR " random allocation" ) )

***Article title***

A scoping review on information and support needs regarding Cancer and Work: Part II – needs of employers and professionals in supporting cancer survivors

***Journal***

Journal of Cancer Survivorship

***Authors***

J.M. Mollet^1,2^, M.A. Greidanus^1,2^, S.F.A. Duijts^3,4,5^, A.G.E.M. de Boer ^1,2,5^, P.M.V Janse^1,2^, I.L. Bakker^1,2^, S.J. Tamminga^1,2^

***Affiliation***

1: Amsterdam UMC location University of Amsterdam, Department of Public and Occupational Health, Amsterdam, The Netherlands

2: Amsterdam Public Health research institute, Societal Participation and Health, Amsterdam, The Netherlands

3: Netherlands Comprehensive Cancer Organization (IKNL), Research and Development, Utrecht, Netherlands

4: Amsterdam UMC location Vrije Universiteit, Department of Medical Psychology, Amsterdam, The Netherlands

5: Cancer Center Amsterdam, Cancer Treatment and Quality of Life, Amsterdam, The Netherlands

***Corresponding author***

Name: Judith Mollet

E-mail address: j.m.mollet@amsterdamumc.nl
