# Supplementary material for: A scoping review on information and support needs regarding cancer and work: part II—needs of employers and professionals in supporting cancer survivors
Source: J Cancer Surviv. 2026 Apr 16;20(Suppl 2):2472–85. doi: 10.1007/s11764-026-02019-z (PMC13744526; doi:10.1007/s11764-026-02019-z)
Supplement: Supplementary file 2 [file 11764_2026_2019_MOESM2_ESM.docx]

**Supplementary file B – characteristics and extracted data**

**Table 1: Study characteristics of the included studies**

| **#** | **Author(s) (year)** | **Country** | **Year(s) of data collection** | **Study design** | | | | |
| --- | --- | --- | --- | --- | --- | --- | --- | --- |
|  |  |  |  | **Cross-sectional** | **Longitudinal** | **Quantitative** | **Qualitative** | |
|  |  |  |  |  |  | Questionnaire | Interview | Focus group |
| 1. | Algeo et al. (2022) | Ireland | 2019 | x |  |  | x |  |
| 2. | Bennink et al. (2021) | Netherlands | 2020 | x |  |  | x |  |
| 3. | Bilodeau et al. (2024) | Canada | 2019-2020 | x |  |  | x |  |
| 4. | Boelhouwer et al. (2021) | Netherlands | 2019-2020 | x |  |  | x |  |
| 5. | Chow et al. (2015) | Malaysia | NR | x |  |  | x |  |
| 6. | De Boer et al. (2017) | Netherlands | NR | x |  |  | x | x |
| 7. | De Rijk et al. (2020) | Netherlands | 2016-2017 | x |  |  | x |  |
| 8. | Désiron et al. (2022) | Belgium | NR | x |  |  | x | x |
| 9. | Désiron et al. (2015) | Belgium | 2008-2009 & 2011 | x |  |  | x | x |
| 10. | Fassier et al. (2018) | France | NR | x |  |  | x | x |
| 11. | Fitch and Nicoll (2019) | Canada | NR | x |  | x | x | x |
| 12. | Greidanus et al. (2021) | Netherlands | 2019 | x |  | x | x |  |
| 13. | Grus et al. (2019) | United States | 2017-2018 | x |  |  | x |  |
| 14. | Kemp et al. (2021) | Australia | NR | x |  |  | x | x |
| 15. | Klaver et al. (2020) | Netherlands | NR | x |  |  |  | x |
| 16. | Knott et al. (2014) | Australia | NR | x |  |  | x | x |
| 17. | Lamort-Bouche et al. (2020) | France | 2015-2016 | x |  |  | x |  |
| 18. | Leensen et al. (2018) | Netherlands | NR |  | x | x | x |  |
| 19. | MacLennan et al. 2017 | United Kingdom | NR | x |  |  | x |  |
| 20. | Marinas-Sanz et al. (2023) | Spain | 2021 | x |  |  |  | x |
| 21. | Martin et al. (2022) | France | 2019 | x |  |  | x |  |
| 22. | Olischlager et al. (2023) | Netherlands | 2021 | x |  |  | x |  |
| 23. | Petersen et al. (2019) | Denmark | 2015-2016 | x |  |  | x |  |
| 24. | Popa et al. (2020) | Romania | 2016 | x |  |  | x |  |
| 25. | Rondinone et al. (2023) | Italy | NR | x |  | x |  |  |
| 26. | Sarfo et al. (2023) | Netherlands | 2016-2019 | x |  |  |  | x |
| 27. | Sheppard et al. (2023) | Australia | 2019 | x |  |  | x | x |
| 28. | Shim et al. (2019) | Korea | 2015-2016 | x |  | x |  |  |
| 29. | Stergiou-Kita et al. (2016)^1, ×^ | Canada | NR | x |  |  | x |  |
| 30. | Stergiou-Kita et al. (2016)^2, ×^ | Canada | NR | x |  |  | x |  |
| 31. | Tamminga et al. (2016) | Netherlands | 2013 | x |  |  | x |  |
| 32. | Tiedtke et al. (2020) | Belgium | NR | x |  |  | x |  |
| 33. | Tiedtke et al. (2017) | Netherlands | NR | x |  |  | x |  |
| 34. | Tiedtke et al. (2014) | Belgium | NR | x |  |  | x |  |
| 35. | Zaman et al. (2017) | Netherlands | 2012-2013 | x |  |  | x |  |
| 36. | Zaman et al. (2020)^3^ | Netherlands | 2015-2018 |  | x | x |  |  |
| 37. | Zaman et al. (2020)^4^ | Netherlands | 2017-2018 | x |  | x |  |  |
| 38. | Zaman et al. (2018) | Netherlands | 2014 | x |  | x | x |  |
| 39. | Zegers et al. (2023) | Netherlands | 2021-2022 | x |  |  | x |  |
| 40. | Zegers et al. (2022) | Netherlands | NR | x |  |  | x |  |

^1^ = Stergiou-Kita et al. (2016): The provision of workplace accommodations following cancer: survivor, provider, and employer perspectives

^2^ = Stergiou-Kita et al. (2016): Am I ready to return to work? Assisting cancer survivors to determine work readiness

^3^ = Zaman et al. (2020): Process evaluation of a tailored work-related support intervention for patients diagnosed with gastrointestinal cancer

^4^ = Zaman et al. (2020): Is a tailored work-related support intervention feasible in everyday clinical practice? The experience of healthcare professionals and patients with cancer

**Table 2: Population characteristics of the included studies**

| **#** | **Author(s) (year)** | **Target group** | **Participants(N)** | **Gender** **(N (% female))** | **Age** **(mean (SD), range)** | **Occupation** |
| --- | --- | --- | --- | --- | --- | --- |
| 1. | Algeo et al. (2022) | Professionals | 15 | NR | NR | Various (e.g., physical therapist, psychologist, other) |
|  |  | Employers | 9 | NR | NR | Various (e.g., HRM, manager, occupational health nurse) |
| 2. | Bennink et al. (2021) | Professionals | 15 | NR | NR | Various (e.g., oncology specific occupational physician, hematologist, patient support, social worker) |
| 3. | Bilodeau et al. (2024) | Employers | 13 | 10 (77%) | NR (NR), NR-NR | Various (e.g., manager, HRM manager, executive manager) |
| 4. | Boelhouwer et al. (2021) | Employers | 11 | 6 (55%) | NR | Various (e.g., manager, director, professor) |
|  |  | Professionals | 47 | 42 (89%) | NR | Various (e.g., reintegration consultant, coach, HRM manager, occupational physician) |
| 5. | Chow et al. (2015) | Professionals | 12 | NR | 48 (NR), NR-NR | NR |
| 6. | De Boer et al. (2017) | Employers | 5 | 5 (100%) | 50 (NR), NR-NR | Various (companies, e.g., manufacturing company, cleaning company, reintegration company) |
| 7. | De Rijk et al. (2020) | Employers | 27 | 10 (37%) | 48 (NR), 28-60 | Various (e.g., HRM manager, supervisor, director) |
| 8. | Désiron et al. (2022) | Professionals | 104 | NR | NR | Various (e.g., nurse, social worker, dokter) |
| 9. | Désiron et al. (2015) | Professionals | 46 | NR | NR | Various (e.g., nurse, occupational therapist, staff members) |
| 10. | Fassier et al. (2018) | Employers | 9 | NR | NR | HRM director and supervisor |
|  |  | Professionals | 82 | NR | NR | Various (e.g., general practitioner, rehabilitation worker, oncologist) |
| 11. | Fitch and Nicoll (2019) | Employers | 19 | NR | NR | NR |
| 12. | Greidanus et al. (2021) | Employers | 22 | 11 (50%) | NR | Various (e.g., HR manager, direct supervisor, re-integration coach) |
| 13. | Grus et al. (2019) | Employers | 4 | 3 (75%) | NR | HR manager and compensation and benefits manager) |
| 14. | Kemp et al. (2021) | Professionals | 41 | 27 (66%) | NR | Primary health care and oncology care professionals |
| 15. | Klaver et al. (2020) | Professionals | 15 | 13 (87%) | 47 (11), 29-67 | Various (e.g., HR consultant, psychologist, occupational therapist) |
| 16. | Knott et al. (2014) | Professionals | 21 | 12 (57%) | NR | Various (e.g., nurse, general practitioner, medical oncologist) |
| 17. | Lamort-Bouche et al. (2020) | Professionals | 20 | 12 (60%) | NR (NR), NR-NR | Various (e.g., medical oncologist, radiation therapy oncologist, gynecological surgeon) |
| 18. | Leensen et al. (2018) | Professionals | 13 | NR | NR | Various (e.g., physical therapist sports physician, oncology specific occupational physician) |
| 19. | MacLennan et al. 2017 | Professionals | 10 | 5 (50%) | 48 (11), NR-NR | Various (e.g., nurse specialist, consultant urologist, oncologist) |
|  |  | Employers | 10 | 4 (40%) | 45 (10), NR-NR | Various (e.g., HRM, occupational health physician, occupational health medical director) |
| 20. | Marinas-Sanz et al. (2023) | Professionals | 8 | NR | NR | Various (e.g., specialists in primary care, breast surgery, gynecology) |
|  |  | Employers | 7 | 6 (86%) | NR | HRM manager and occupational risk prevention |
| 21. | Martin et al. (2022) | Professionals | 18 | NR | 45 (NR), 32–60 | Various (e.g., oncologist, psychologist, social worker) |
| 22. | Olischlager et al. (2023) | Professionals | 9 | 4 (44%) | 55 (7), 43-70 | Various (e.g., oncologist, representative of patient organization, oncology specific occupational physician) |
| 23. | Petersen et al. (2019) | Employers | 7 | 6 (86%) | NR (NR), 38-57 | Direct supervisor |
| 24. | Popa et al. (2020) | Employers | 20 | 13 (65%) | NR | Various (e.g., HR (deputy) director, HR counsellor, team supervisor |
| 25. | Rondinone et al. (2023) | Professionals | 157 | 55 (35%) | NR | Occupational health physician |
| 26. | Sarfo et al. (2023) | Professionals | 27 | 13 (48%) | 53 (NR), 41-62 | General practitioner and occupational health physician |
| 27. | Sheppard et al. (2023) | Professionals | 8 | NR | NR | Reintegration advisor |
| 28. | Shim et al. (2019) | Professionals | 44 | 5 (11%) | NR | Occupational health physician |
| 29. | Stergiou-Kita et al. (2016)^1, ×^ | Professionals | 16 | NA | 48 (NR), 29-62 | Various (e.g., rehabilitation consultant, occupational therapist, psychologist) |
|  |  | Employers | 8 | NA | 48 (NR), 32-63 | Various (e.g., manager, rehabilitation team leader, HR manager) |
| 30. | Stergiou-Kita et al. (2016)^2, ×^ | Professionals | 16 | NA | 48 (NR), 29-62 | Various (e.g., rehabilitation consultant, occupational therapist, psychologist, HR manager) |
| 31. | Tamminga et al. (2016) | Employers | 11 | NR | NR | NR |
|  |  | Professionals | 22 | NR | NR | Occupational health physician, general practitioner and social worker |
| 32. | Tiedtke et al. (2020) | Employers | 11 | 4 (33%) | NR | Operations manager and HRM |
| 33. | Tiedtke et al. (2017) | Employers | 30 | 18 (60%) | NR | Supervisor and HR manager |
| 34. | Tiedtke et al. (2014) | Employers | 17 | 10 (59%) | NR | HR manager, department manager, director |
| 35. | Zaman et al. (2017) | Professionals | 13 | 6 (46%) | 55 (NR), 31-65 | Oncology specific occupational physician |
| 36. | Zaman et al. (2020)^3^ | Professionals | 40 | NR | NR | Specialized oncology nurse and oncology specific occupational physician |
| 37. | Zaman et al. (2020)^4^ | Professionals | 18 | NR | NR | Specialized oncology nurse and oncology specific occupational physician |
| 38. | Zaman et al. (2018) | Professionals | 44 | NR | NR | Various (e.g., gastrointestinal oncologists/surgeons, gastrointestinal oncology nurses, social workers) |
|  |  | Professionals | 12 | NR | NR | Various (e.g., gastrointestinal oncologists/surgeons, gastrointestinal oncology nurses, oncological occupational physicians) |
| 39. | Zegers et al. (2023) | Professionals | 16 | NR | 49 (8), NR-NR | Various (e.g., nurse, physician, legal advisor) |
| 40. | Zegers et al. (2022) | Professionals | 17 | 11 (65%) | 52 (9), NR | Various (e.g., reintegration consultant, oncology specific occupational physician, clinical neuropsychologist) |

| **#** | **Author(s) (year)** | **Target group** | **Participants(N)** | **Gender** **(N (% female))** | **Age** **(mean (SD), range)** | **Occupation** |
| --- | --- | --- | --- | --- | --- | --- |
| 1. | Algeo et al. (2022) | Professionals | 15 | NR | NR | Various (e.g., physical therapist, psychologist, other) |
|  |  | Employers | 9 | NR | NR | Various (e.g., HRM, manager, occupational health nurse) |
| 2. | Bennink et al. (2021) | Professionals | 15 | NR | NR | Various (e.g., oncology specific occupational physician, hematologist, patient support, social worker) |
| 3. | Bilodeau et al. (2024) | Employers | 13 | 10 (77%) | NR (NR), NR-NR | Various (e.g., manager, HRM manager, executive manager) |
| 4. | Boelhouwer et al. (2021) | Employers | 11 | 6 (55%) | NR | Various (e.g., manager, director, professor) |
|  |  | Professionals | 47 | 42 (89%) | NR | Various (e.g., reintegration consultant, coach, HRM manager, occupational physician) |
| 5. | Chow et al. (2015) | Professionals | 12 | NR | 48 (NR), NR-NR | NR |
| 6. | De Boer et al. (2017) | Employers | 5 | 5 (100%) | 50 (NR), NR-NR | Various (companies, e.g., manufacturing company, cleaning company, reintegration company) |
| 7. | De Rijk et al. (2020) | Employers | 27 | 10 (37%) | 48 (NR), 28-60 | Various (e.g., HRM manager, supervisor, director) |
| 8. | Désiron et al. (2022) | Professionals | 104 | NR | NR | Various (e.g., nurse, social worker, dokter) |
| 9. | Désiron et al. (2015) | Professionals | 46 | NR | NR | Various (e.g., nurse, occupational therapist, staff members) |
| 10. | Fassier et al. (2018) | Employers | 9 | NR | NR | HRM director and supervisor |
|  |  | Professionals | 82 | NR | NR | Various (e.g., general practitioner, rehabilitation worker, oncologist) |
| 11. | Fitch and Nicoll (2019) | Employers | 19 | NR | NR | NR |
| 12. | Greidanus et al. (2021) | Employers | 22 | 11 (50%) | NR | Various (e.g., HR manager, direct supervisor, re-integration coach) |
| 13. | Grus et al. (2019) | Employers | 4 | 3 (75%) | NR | HR manager and compensation and benefits manager) |
| 14. | Kemp et al. (2021) | Professionals | 41 | 27 (66%) | NR | Primary health care and oncology care professionals |
| 15. | Klaver et al. (2020) | Professionals | 15 | 13 (87%) | 47 (11), 29-67 | Various (e.g., HR consultant, psychologist, occupational therapist) |
| 16. | Knott et al. (2014) | Professionals | 21 | 12 (57%) | NR | Various (e.g., nurse, general practitioner, medical oncologist) |
| 17. | Lamort-Bouche et al. (2020) | Professionals | 20 | 12 (60%) | NR (NR), NR-NR | Various (e.g., medical oncologist, radiation therapy oncologist, gynecological surgeon) |
| 18. | Leensen et al. (2018) | Professionals | 13 | NR | NR | Various (e.g., physical therapist sports physician, oncology specific occupational physician) |
| 19. | MacLennan et al. 2017 | Professionals | 10 | 5 (50%) | 48 (11), NR-NR | Various (e.g., nurse specialist, consultant urologist, oncologist) |
|  |  | Employers | 10 | 4 (40%) | 45 (10), NR-NR | Various (e.g., HRM, occupational health physician, occupational health medical director) |
| 20. | Marinas-Sanz et al. (2023) | Professionals | 8 | NR | NR | Various (e.g., specialists in primary care, breast surgery, gynecology) |
|  |  | Employers | 7 | 6 (86%) | NR | HRM manager and occupational risk prevention |
| 21. | Martin et al. (2022) | Professionals | 18 | NR | 45 (NR), 32–60 | Various (e.g., oncologist, psychologist, social worker) |
| 22. | Olischlager et al. (2023) | Professionals | 9 | 4 (44%) | 55 (7), 43-70 | Various (e.g., oncologist, representative of patient organization, oncology specific occupational physician) |
| 23. | Petersen et al. (2019) | Employers | 7 | 6 (86%) | NR (NR), 38-57 | Direct supervisor |
| 24. | Popa et al. (2020) | Employers | 20 | 13 (65%) | NR | Various (e.g., HR (deputy) director, HR counsellor, team supervisor |
| 25. | Rondinone et al. (2023) | Professionals | 157 | 55 (35%) | NR | Occupational health physician |
| 26. | Sarfo et al. (2023) | Professionals | 27 | 13 (48%) | 53 (NR), 41-62 | General practitioner and occupational health physician |
| 27. | Sheppard et al. (2023) | Professionals | 8 | NR | NR | Reintegration advisor |
| 28. | Shim et al. (2019) | Professionals | 44 | 5 (11%) | NR | Occupational health physician |
| 29. | Stergiou-Kita et al. (2016)^1, ×^ | Professionals | 16 | NA | 48 (NR), 29-62 | Various (e.g., rehabilitation consultant, occupational therapist, psychologist) |
|  |  | Employers | 8 | NA | 48 (NR), 32-63 | Various (e.g., manager, rehabilitation team leader, HR manager) |
| 30. | Stergiou-Kita et al. (2016)^2, ×^ | Professionals | 16 | NA | 48 (NR), 29-62 | Various (e.g., rehabilitation consultant, occupational therapist, psychologist, HR manager) |
| 31. | Tamminga et al. (2016) | Employers | 11 | NR | NR | NR |
|  |  | Professionals | 22 | NR | NR | Occupational health physician, general practitioner and social worker |
| 32. | Tiedtke et al. (2020) | Employers | 11 | 4 (33%) | NR | Operations manager and HRM |
| 33. | Tiedtke et al. (2017) | Employers | 30 | 18 (60%) | NR | Supervisor and HR manager |
| 34. | Tiedtke et al. (2014) | Employers | 17 | 10 (59%) | NR | HR manager, department manager, director |
| 35. | Zaman et al. (2017) | Professionals | 13 | 6 (46%) | 55 (NR), 31-65 | Oncology specific occupational physician |
| 36. | Zaman et al. (2020)^3^ | Professionals | 40 | NR | NR | Specialized oncology nurse and oncology specific occupational physician |
| 37. | Zaman et al. (2020)^4^ | Professionals | 18 | NR | NR | Specialized oncology nurse and oncology specific occupational physician |
| 38. | Zaman et al. (2018) | Professionals | 44 | NR | NR | Various (e.g., gastrointestinal oncologists/surgeons, gastrointestinal oncology nurses, social workers) |
|  |  | Professionals | 12 | NR | NR | Various (e.g., gastrointestinal oncologists/surgeons, gastrointestinal oncology nurses, oncological occupational physicians) |
| 39. | Zegers et al. (2023) | Professionals | 16 | NR | 49 (8), NR-NR | Various (e.g., nurse, physician, legal advisor) |
| 40. | Zegers et al. (2022) | Professionals | 17 | 11 (65%) | 52 (9), NR | Various (e.g., reintegration consultant, oncology specific occupational physician, clinical neuropsychologist) |

^1^ = Stergiou-Kita et al. et al. (2016): The provision of workplace accommodations following cancer: survivor, provider, and employer perspectives

^2^ = Stergiou-Kita et al. (2016): Am I ready to return to work? Assisting cancer survivors to determine work readiness

^3^ = Zaman et al. (2020): Process evaluation of a tailored work-related support intervention for patients diagnosed with gastrointestinal cancer

^4^ = Zaman et al. (2020): Is a tailored work-related support intervention feasible in everyday clinical practice? The experience of healthcare professionals and patients with cancer

^×^ = Both studies examine the same population of professionals

NR = Not reported

**Table 3a: Identified information needs of professionals**

| **#** | **Authors (year)** | **Extracted from full text** | **Information need** |
| --- | --- | --- | --- |
| 1. | Algeo et al. (2022) | Women with breast cancer and healthcare professional cohorts shared a reduced awareness of  employment rights, particularly pertaining to cancer diagnoses; | Need to be aware of the legal obligation on employers to offer accommodations |
| 2. | Bennink et al. (2021) | NR | NR |
| 4. | Boelhouwer et al. (2021) | one of the case managers expresses the need for knowledge about how to adapt specific work tasks to a less demanding cognitive level. | Need for knowledge about how to adapt specific work tasks to a less demanding cognitive level. |
| 5. | Chow et al. (2015) | Some HCP did not fully understand their role and scop of duties, yet this could help the RTW process | Need to fully understand their role and scope of duties |
| 8. | Desiron et al. (2022) | The participants were aware that they lacked knowledge about the legal and regulatory framework for occupational reintegration. This lack of up-to-date knowledge is particularly frustrating for the social workers but also for the other participants, and it has a negative impact on the quality of care they can provide. | Need for knowledge about the legal and regulatory framework for occupational reintegration. |
|  |  | It was also mentioned several times that the lack of knowledge about the competences of other professionals led to a form of compartmentalization. | Need for knowledge about the competences of other professionals. |
| 9. | Desiron et al. (2015) | For all the OT department heads interviewed, an in-depth knowledge of the values, motivation and habits of the patient is of primary importance for the success of a rehabilitation programme, regardless of their medical problem. They therefore stress the importance of a broad and yet detailed intake in order to construct a clear knowledge of the patients' situation, norms, values, habituations activities and participation. | Need for knowledge of the values, motivation and habits of the patients for rehabilitation to be successful |
| 10. | Fassier et al. (2018) | Oncologists had different levels of commitment toward the work issues of their patients. All declared having insufficient knowledge about work. They did not know how to help their patients when they expressed their concerns about resuming work. Oncologists mostly emphasized the importance of individual characteristics of the women (motivation and temper) in returning to work. | Need for knowledge about (e.g. to know how to help their patients when they expressed their concerns about resuming work / individual characteristics of the women (motivation and temper) in returning to work). |
|  |  | Although all the SIPs felt concerned in helping women with breast cancer to RTW, they reported some limitations about their possibilities. Contrary to other medical conditions such as low back pain, they mentioned lacking guidelines to appreciate the risk of long-term work absence and disability after breast cancer. They had limited possibilities to exchange information with their social department due to legal constraints related to medical secrecy and privacy of personal data. | Social insurance physicians need guidelines to appreciate the risk of long-term work absence and disability after breast cancer. |
|  |  | Social workers: They reported difficulties to set standard criteria to identify socially deprived women. Rather, they insisted on the uniqueness of each situation and the need to consider the interactions of many factors such as financial resources, geographic isolation, social isolation, etc. | Social workers need a set of standard criteria to identify socially deprived women (e.g. interactions of many factors such as financial resources, geographic isolation, social isolation, etc) |
| 14. | Kemp et al. (2021) | NR | NR |
| 15. | Klaver et al. (2020) | NR | NR |
| 16. | Knott et al. (2014) | NR | NR |
| 17. | Lamort-Bouche et al. (2020) | Many participants shared concerns about the “right time and right way” to address RTW with their patients given the variety of individual situations. “  We don't really know how to situate return to work during patient care, (...) well, I find it difficult to decide when to talk about return to work and what resources are available and already set up to help the patients with that.” (Medical oncologist) | Need for information on when to talk about return to work and what resources are available and already set up to help the patients with that. |
|  |  | The main barriers to the involvement of BC specialists in their patients' RTW issues included (i) lack of time, (ii) lack of knowledge, especially regarding RTW procedures and legislation, (iii) the potential barriers to RTW, (iv) the role of the various actors involved in the RTW process, particularly GP and OP, (v) lack of awareness of the importance of work to their patients, and (vi) a professional attitude exclusively focused on care and cure objectives | Need for knowledge on the importance of work for the patient. |
| 18. | Leensen et al. (2018) | The barriers experienced in conducting the SMA were insufficient knowledge about physical complaints resulting from cancer treatment | Need for knowledge about the physical complaints resulting from cancer treatment. |
| 19. | MacLennan et al. (2017) | Collectively, participants recognised that there is a need for a better system for providing work-related information, advice and related support to people with urological cancer. | Need for a better system for providing work-related information, advice and related support to people with urological cancer |
|  |  | Many demonstrated a lack of awareness of possible information, advice and related support resources. | Need for awareness of possible information, advice and related support resources. |
|  |  | This was related to the need to develop a clear understanding of who should be doing what and when. | Need to develop a clear understanding of who  should be doing what and when |
| 20. | Marinas-Sanz et al. (2023) | The importance of multidisciplinary collaboration was unanimously underlined and considered essential. However, Occupational Medicine physicians manifested they are excluded from the health system not having access to all data related to treatment and clinical evolution of the patient making that collaboration difficult | Need for having access to all data related to treatment and clinical evolution of the patient so that multidisciplinary collaboration is possible |
| 21. | Martin et al. (2022) | Besides an important lack of knowledge about the broad topic of RTW, | Need for knowledge about the broad topic of return to work |
|  |  | providers also declared a lack of knowledge about existing interventions to help patients RTW | Need for knowledge about existing interventions to help patients return to work |
|  |  | Preferences and needs regarding interventions to help patients RTW: A place to find more information about work | Need to have a place to find more information about work |
| 22. | Olischlager et al. (2023) | People really feel like they have to do it [i.e., moving through the health care system and through the re-integration process] on their own. But their employers face the same problem, and health care professionals as well. They also do not know how to deal with rare cancer and work. | Need for information how to move through the re-integration process and know how to deal with rare cancer and work. |
| 25. | Rondinone et al. (2023) | This paragraph shows results on information demands and updating training needs related to the management of WAC, by the OP’s point of view. 42.0% of the sample agreed (mostly and completely) with the statement that specific training in the field of cancer and work is important for the OP. On a 0–10 scale (0 = useless; 10 = completely useful), a list of topics that would be necessary for the training needs in terms of neoplastic diseases and work is shown in decreasing order, from the most useful to the least useful. The most useful are criteria for formulating the fitness judgment (6.86), the health surveillance protocols (5.96) and the reasonable accommodations (5.58) (Fig 1). | Need for information and training for occupational physicians about cancer and work such as criteria for formulating the fitness judgment, the health surveillance protocols and the reasonable accommodations. |
| 26. | Sarfo et al. (2023) | GPs said that it is not always clear to them when one of their patients has finished their cancer treatment. GPs stated that although they receive letters from oncology specialists about the cancer trajectory, it is not clear at what point GPs are intended to be involved in cancer aftercare. GPs mentioned that it would be helpful to establish both their own role and that of the oncological specialist more clearly, including who should be tasked with work guidance and at what moment. | General practitioners need information from the oncology specialist about when treatment is finished and need to know when they are intended to be involved in cancer aftercare. |
|  |  |  | Need to establish both their own role and that of the oncological specialist more clearly, including who should be tasked with work guidance and at what moment. |
| 27. | Sheppard et al. (2023) | The effectiveness of the consultant training; some found themselves in situations where they felt underprepared, especially regarding emotionally-laden or sensitive topics | Need for being better prepared for consultant training, especially regarding emotionally-laden or sensitive topics |
| 28. | Shim et al. (2019) | Approximately 25% did not agree with the statement: “I have access to sufficient information regarding the potential psychological impact of a cancer diagnosis and its subsequent treatment to comment on managing such issues as part of a return-to-work plan. | Need to have sufficient information regarding the potential psychological impact of a cancer diagnosis and its subsequent treatment to comment on managing such issues as part of a return-to-work plan |
|  |  | Approximately 57% did not agree with the statement: “Doctors of employees with common cancers have good insight into the role of OH in supporting employees during periods of treatment and subsequent return to work | Medical physicians need to be better informed about the role of occupational health in supporting employees during periods of treatment and subsequent return to work |
|  |  | Approximately 45% did not agree with the statement: Doctors of employees with common cancers have good insight into the nature of the information required by employers to help identify the most appropriate occupational outcome for their patients.  25% disagree: My training in OHP has provided me with the appropriate communication skills to comfortably discuss issues of prognosis and functional outcomes with patients with cancer | Medical physicians need to be better informed about the nature of the information required by employers to help identify the most appropriate occupational outcome for their patients. |
|  |  |  | Need for communication skills training to comfortably discuss issues of prognosis and functional outcomes with patients with cancer |
|  |  | Measures for overcoming obstacles: Training and materials about the issue for physicians | Need for training and materials |
| 29. | Stergiou-Kita et al. (2016)^1^ | However, despite the importance of appropriate medical documentation, P16 explained how few physicians appeared aware of the weight and importance of their recommendations and how challenging it may be for survivors to explain their accommodation needs: P16: [It is important to] make sure that the individual  [survivor] works with their physician. In many cases, the physician isn’t aware of, or doesn’t understand the pressure being placed on the employee to return to work. Getting the physician into the picture to get a good report [is important for] making sure that the employer or the disability insurer is fully aware of the medical condition… | Need to be aware of the weight and importance of their recommendations and how challenging it may be for survivors to explain their accommodation needs |
| 30. | Stergiou-Kita et al. (2016)^2^ | providers described the importance of gaining an understanding of the particular strengths a survivor was bringing to his/her work, any self-perceived barriers to returning to work, and/ or medically related limitations or restrictions (as identified by medical professionals) that would need to be adhered to. Due to perceived lack of detail in most medical documentation of restrictions, providers reported that they also specifically relied on discussions with survivors of any concerns they may have about returning to work and symptoms they believed may impair their abilities to return to work. | Need for gaining an understanding of the particular strengths a survivor was bringing to his/her work,  any self-perceived barriers to returning to work, and/ or medically related limitations or restrictions (as identified by medical professionals) |
|  |  | The first related to the lack of standardized tools, guidelines, or processes for evaluating work readiness within the current Canadian health care/hospital system. Health care provider 6 explains below that while the clinical templates she uses has a question or two pertaining to work and return to work, little instruction is provided on how to follow-up with survivors who express work-related concerns. Follow-up on work concerns became a matter of discretion, and providers whose training may be more suitably apt to addressing vocational issues (e.g., occupational therapy) are more likely to provide additional followup care. | Need for standardized tools, guidelines, or processes for evaluating work readiness within the current Canadian health care/ hospital system. |
|  |  | The second challenge related to inconsistencies in health care providers’ understanding of how the complexity of cancer could impact their work abilities. This could in turn result in similar recommendations made for all cancer survivors or inconsistent messaging regarding work readiness between different health care providers as illustrated below: | Need for understanding of how the complexity of cancer could impact their work abilities |
| 31. | Tamminga et al. (2016) | Thus, the occupational physicians in particular indicated that more detailed information was required to make the intervention useful. | Need for detailed information |
| 35. | Zaman et al. (2017) | NR | NR |
| 36. | Zaman et al. (2020)^3^ | NR | NR |
| 37. | Zaman et al. (2020)^4^ | NR | NR |
| 38. | Zaman et al. (2018) | NR | NR |
| 39. | Zegers et al. (2023) | Professionals such as occupational physicians stated that, if they were to be better informed about the medical details of patients with a malignant glioma (e.g., received relevant medical data), they would be better able to assess patients’ work ability and therefore tailor their advice. | Need to have medical details of patients with a malignant glioma (e.g., received relevant medical data) |
|  |  | Most professionals mentioned that they lacked knowledge regarding Dutch laws and regulations concerning sick leave, work disability, and reintegration. | Need to have knowledge regarding Dutch laws and regulations concerning sick leave, work disability, and reintegration |
|  |  | These professionals typically referred patients elsewhere (e.g., to social workers or to professionals in the patient’s workplace), most felt unsure as to who they could best refer their patients to. | Need to have information on who they could best refer their patients to. |
| 40. | Zegers et al. (2022) | Participants indicated a lack of knowledge among various professionals, including themselves, regarding the legislative aspects of reintegration (e.g., sickness absence and work disability legislation). According to most, a lack of legislative knowledge was mainly seen among health care professionals and employers. Rather, participants indicated that having a better understanding of current laws and regulations creates opportunities for customisation for employees with advanced cancer. | Need to have information on the legislative aspects of reintegration (e.g., sickness absence and work disability legislation). |
|  |  | According to participants, the hesitancy to communicate about work with a patient with advanced cancer could also stem from a lack of relevant knowledge, for example, concerning the consequences of the disease/treatment or relevant laws and regulations regarding sickness absence and reintegration. | Need to have knowledge, for example, concerning the consequences of the disease/treatment or relevant laws and regulations regarding sickness absence and reintegration. |
|  |  | Yet, these professionals were often said to lack (advanced) oncology-related knowledge to support patients in a targeted and personalised way. | Need to have (advanced) oncology-related knowledge to support patients in a targeted and personalized way. |

^1^ = Stergiou-Kita et al. et al. (2016): The provision of workplace accommodations following cancer: survivor, provider, and employer perspectives

^2^ = Stergiou-Kita et al. (2016): Am I ready to return to work? Assisting cancer survivors to determine work readiness

^3^ = Zaman et al. (2020): Process evaluation of a tailored work-related support intervention for patients diagnosed with gastrointestinal cancer

^4^ = Zaman et al. (2020): Is a tailored work-related support intervention feasible in everyday clinical practice? The experience of healthcare professionals and patients with cancer

NR = Not reported

**Table 3b:** **Identified information needs of professionals**

| **#** | **Authors (year)** | **Extracted from full text** | **Information need** |
| --- | --- | --- | --- |
| 2. | Algeo et al. (2022) | Some participants suggested that this reflects poor awareness among colleagues/employers of long-term cancer side-effects | Need to be aware of long-term cancer side-effects |
| 3. | Bilodeau et al. (2024) | a widely reported issue by our participants was the need to be adequately equipped with information regarding the BCSs’ work abilities and the late effects of cancer. | Need to be adequately equipped with information regarding the cancer survivors’ work abilities and the late effects of cancer. |
| 4. | Boelhouwer et al. (2021) | NR | NR |
| 6. | De Boer et al. (2017) | NR | NR |
| 7. | De Rijk et al. (2020) | After the cancer diagnosis is disclosed, the employer—mostly the line manager—begins to obtain relevant information needed for early decision-making, either in the same meeting during which the disclosure took place or in additional meetings. In some countries, an occupational physician meets the worker and delivers the information needed. The aim of this phase is to gain knowledge on the functional consequences of medical situation, the treatment and the psychosocial situation. | Need for knowledge on functional consequences of medical situation, treatment and psychosocial situation. |
|  |  | All employers try to keep in touch with their affected workers in different ways and with different frequencies. There might be meetings at home or in the organisation, or telephone calls. The line manager, HR professionals, or occupational physicians might be involved. The goals of this problem solving includes staying in touch with the worker in order to ensure that a worker’s needs are clear and to collect necessary information on the course of the illness and the treatment via the worker. | Need to collect necessary information on the course of illness and treatment. |
| 10. | Fassier et al. (2018) | Information and training to support a woman returning to work after cancer | Need for information and training to support a woman returning to work after cancer |
| 11. | Fitch and Nicoll (2019) | NR | NR |
| 12. | Greidanus et al. (2021) | Participants were motivated to use the intervention by intrinsic as well as extrinsic factors. As to the intrinsic facilitators, participants indicated that they felt responsible and were motivated to provide the best possible support to the cancer survivor:  I’m just very intrinsically motivated to support colleagues, in any form whatsoever (…) and if you are diagnosed with cancer (…) that simply does a lot with somebody. I really want as many tools as possible (…) to get someone through that | Need for information on how to provide the best possible support to cancer survivors, tailored to the specific situation of their cancer survivor (e.g. in terms of return to work phase) |
|  |  | A few additions to the intervention were suggested by participants, to enhance its usefulness or usability: a clear introduction or overview on the homepage, general information about cancer and national legislations, suggestions for the allocation of tasks within the organization (e.g. between the HR manager, direct supervisor and occupational physician), and a forum offering the possibility to consult with fellow employers. | Need for general information about cancer. |
|  |  |  | Need for general information about national legislations. |
|  |  |  | Need for suggestions for the allocation of tasks within the organization (e.g. between  the HR manager, direct supervisor and occupational physician). |
|  |  |  | Need for a forum offering the possibility to consult with fellow employers. |
|  |  | Nevertheless, the phases were not adequate for all situations, and the participants suggested adding a 5th phase to the intervention:  …the question is whether [this cancer survivor] will survive. So, those are really tough things. Then it does not make sense to say, well, how are we going to plan your return to work. But you are still the manager and it is your task to, let’s say, guide this in a different way, and I miss that a little in the [intervention]. | Need for information on how to provide support to cancer survivors who will not be able to return to work and/or will not survive. |
|  |  | Some participants indicated that they missed a particular type of cancer survivor, for example a non-cooperative cancer survivor who wants to slow down the RTW or an even more emotional cancer survivor. | Need for information on how to support non-cooperative cancer survivors who want to slow down the RTW, or more emotional cancer survivor. |
| 13. | Grus et al. (2019) | NR | NR |
| 19. | MacLennan et al. (2017) | There was a need to develop a clear understanding of who  should be doing what and when | Need to develop a clear understanding of who should be doing what and when |
|  |  | need for strategic information such as that about working after cancer, adaptation to work and workplace accommodations, disclosure and practical information on how to help individuals in the workplace and where to access specialist support | Need for strategic information such as that about working after cancer, adaptation to work and workplace accommodations, disclosure and practical information on how to help individuals in the workplace and where to access specialist support |
| 20. | Marinas-Sanz et al. (2023) | The group agreed about the need to create a legal framework that adapts to different situations and guarantees job stability after the period of temporary incapacity, while giving companies the support to deal with these situations | Need for a legal framework that adapts to different situations and guarantees job stability after the period of temporary incapacity, while giving companies the support to deal with these situations |
|  |  | Employers pointed out that while their companies had a welcome protocol, there was no return-to-work protocol although it would be useful. | Need for return-to-work protocol |
| 23. | Petersen et al. (2019) | A recurring theme voiced by employer was inadequate knowledge of how to manage the RTW process. Even though the employers did have contact with social workers at the municipal job centers as part of the RTW intervention study, they experienced that contact with job centers in general did not support the management of the RTW process of sick-listed employees. When asked about their contact with the municipal social workers responsible for the employee’s RTW, the employers emphasized that the RTW plan was drawn up by the social workers in collaboration with the employee undergoing cancer treatment with a view to meet their needs, not theirs as employers | Need for information about how to manage the return to work process, in which not only the needs of the employees are met but also the needs of the employers |
| 24. | Popa et al. (2020) | NR | NR |
| 29. | Stergiou-Kita et al. (2016)^1^ | Employers also reported frustrations with receiving insufficiently detailed medical information to develop job-specific  accommodations or very poorly articulated (and thus very  difficult to interpret) medical restrictions and limitations | Need to have detailed medical information to develop job-specific accommodations |
| 31. | Tamminga et al. (2016) | The main issue that arose from the interviews was that participants needed more detailed information, mainly on the different cancer diagnoses and treatments, and their possible effects on the employee’s ability to return to work. | Need more detailed information, mainly on the different cancer diagnoses and treatments, and their possible effects on the employee’s ability to return to work. |
| 32. | Tiedtke et al. (2020) | After being informed of the cancer diagnosis and showing their empathy, the employers seemed to be lost and in need of information, especially on rights and duties | Need of information, especially on rights and duties |
|  |  | For example, knowledge of the new reintegration legislation was virtually absent. | Need for knowledge of the new reintegration legislation. |
|  |  | For the employers, a fourth question arose. How to communicate with the (absent) employee and how to inform the colleagues before and after the return? They also had to be aware of the sick employee’s privacy, as noted earlier | Need for information on how to communicate with the (absent) employee and how to inform the colleagues before and after the return. They also had to be aware of the sick employee’s privacy, as noted earlier |
|  |  | Employers mentioned feeling unsure about contacting— and potentially replacing—the absent employee. Since they were concerned and lacked information about the employee’s situation, they were open to learning skills to facilitate communication between the employer, the colleagues and the employee—or a form with a list of questions to ask (do’s and don’ts) | Need to learn learning skills on how to facilitate communication between the employer, the colleagues and the employee—or a form with a list of questions to ask (do’s and don’ts) |
| 33. | Tiedtke et al. (2017) | According to the employers, communication during this phase {employee’s decease)} took an enormous effort, also because of a lack of existing guidelines. | Need to have guidelines. |
|  |  | The employers said that they searched for the right moment to discuss the important matters. For example, when does employability come up for discussion? Was it after treatment, or at the employee’s initiative? Not all employees were able or motivated to return to work after cancer. How to adapt the tasks to the employee without disadvantaging the companies’ interest was another dilemma in this respect. To what extent were they allowed to make demands and push the employee’s boundaries?  Could they confront the employee, who might want to stay active, with the fact that he or she did not perform well enough anymore? Were they allowed to take away responsibilities from the employee concerned? | Need to have information on the right moment to discuss the important matters. Need to have information on how to adapt the tasks to the employee without disadvantaging the companies’ interest. |
|  |  |  | Need to have information on to what extend they were allowed to make demands and push the employee’s boundaries. Need for information on dilemma’s such as Could they confront the employee, who might want to stay active, with the fact that he or she did not perform well enough anymore? Were they allowed to take away responsibilities from the employee concerned? |
|  |  | Finally, the Dutch legislative framework was considered helpful only during the first 2 years, indicating a need for examples of how to deal with longer cases. In case an employee passed away, care had to be given to their close family as well. | Need for examples of how to deal with longer cases. In case an employee passed away, care had to be given to their close family as well. |
| 34. | Tiedtke et al. (2014) | The interviewed employers related how from the moment their employee was absent, they were faced with a number of uncertainties about the diagnosis, | Need to have information about the diagnosis, the treatment, the prognosis and the guidance needed by the employee. |
|  |  | the treatment, the prognosis and the guidance needed by the employee. This uncertainty also seemed to be related to the employer’s lack of experience. What they most wanted was information. The interviews revealed that employers wondered what the employee was going through, what the future held for her and how serious the situation was. How should they help the employee? What questions were they allowed to ask, what not? Should they stay in contact with the sick employee and if so, how? How would the employer know whether the employee appreciated his/her questions and concern? | And answers to questions like: how should they help the employee? What questions were they allowed to ask, what not? Should they stay in contact with the sick employee and if so, how? How would the employer know whether the employee appreciated his/her questions and concern? |
|  |  | If there was no consultation with the company doctor, how would the employer know whether the employee was in a position to fulfil her tasks and cope with the rhythm, pace and content of the job? | Need to have information from the occupational physician on whether the employee was in a position to fulfil her tasks and cope with the rhythm, pace and content of the job |
|  |  | In addition, many employers said that they didn’t have enough information about the legal options concerning RTW, which contributed to their uncertainty. So as not to make any mistakes, they had to go and find out from the HR department | Need to have information about the legal options concerning return to work |
|  |  | Virtually all the employers interviewed experienced this uncertainty and relayed the message that a broader picture of the disease would help them to be able to understand and offer help. | Need to have a broader picture of the disease would help them to be able to understand and offer help. |
|  |  | The way in which the employee communicated about her illness and her RTW also played a role in the employer’s experience. If there was lots of openness, the employer had fewer questions about the course of the illness and he/she felt more certain about his/her approach and in a better position to take the right decisions. | Need to have open communication with the employee |

^1^ = Stergiou-Kita et al. et al. (2016): The provision of workplace accommodations following cancer: survivor, provider, and employer perspectives

NR = Not reported

**Table 4a: Identified support needs of professionals**

| **#** | **Authors (year)** | **Extracted from full text** | **Support need** |
| --- | --- | --- | --- |
| 1. | Algeo et al. (2022) | NR | NR |
| 2. | Bennink et al. (2021) | The interviewed OP acknowledged that counselling MM patients is rare and that specific skills regarding MM is lacking | Occupational physicians need specific skills to council patients with multi myeloma |
| 4. | Boelhouwer et al. (2021) | one of the case managers expresses the need for knowledge about how to adapt specific work tasks to a less demanding cognitive level. | Behoefte aan informatie over aanpassen van werktaken naar een minder veeleisend cognitief niveau |
| 5. | Chow et al. (2015) | NR | NR |
| 8. | Desiron et al. (2022) | Although the analysis reveals the need to assign tasks to care providers who have specific competences in a field, it turns out that participants also consider it important to be able to assign the topic of “work” to a specific care professional who can then be responsible for coordination | Need to assign the topic of “work” to a specific care professional who can then be responsible for coordination. |
|  |  | Participants agreed that a targeted approach to RTW support for occupationally active cancer patients should follow a stepwise plan that is the same for all patients as they progress in their recovery. There was also relative unanimity that such a “common step plan” must allow plenty of space for “tailored work” This should allow for different stages of the common plan to be carried out according to the patient's individual situation | Need for a ‘common step plan’ which allows plenty of space for ‘tailored work'. |
|  |  | The particpants in this study are clearly convinced that collaboration with professionals outside the hospital is necessary to achieve good results | Need to collaborate with professionals outside the hospital to achieve good results. |
| 9. | Desiron et al. (2015) | Also for the focus group members, it is clear that quality of care for BC patients in assisting them to restore their quality of life (including RTW) needs to be delivered by a multidisciplinary team. | Need to work in a multidisciplinary team to answer patient specific needs. |
|  |  | The OT department heads indicate the necessity of a workplace visit to enable the occupational therapist to have a good view of the occupation (the specific activities) of the patient. | Need for a workplace visit to enable the OT to have a good view of the occupation and activities of the patient. |
| 10. | Fassier et al. (2018) | General practitioners expressed that supporting their patients in returning to work was part of their role, but reported lacking of collaboration with social insurance physicians (SIPs) and OPs in this respect. | General practitioners: Need for collaboration with social insurance physicians and occupational physicians. |
| 14. | Kemp et al. (2021) | Some discussed a multidisciplinary approach to return to work assessment, including referral to other specialists such as occupational physicians. | Need for multidisciplinary approach to RTW assessment, e.g. a panel with the GP, a specialist, an occupational therapist and a physiotherapist. |
|  |  | Finally, HCPs discussed the issue of how to assess return to work capacity. While some indicated little difficulty, most agreed there was no specific tool, nor had they received formal training in assessing return to work capacity in cancer survivors. Judgement of survivors’ capacity to perform employment-related tasks was therefore subjective, with assessment consequently depending at times on an on-going relationship with the patient and in-depth knowledge of the requirements of their employment. | Need for support on how to assess return to work capacity of cancer survivors, since it is now subjective since there is no specific tool available at the moment. |
| 15. | Klaver et al. (2020) | Screening: it was suggested that implementation of a screening program for long-term effects of cancer, including cancer-related cognitive problems, might facilitate professionals to offer adequate care for cancer survivors who deal with cancer-related cognitive problems at work. | Needs to improve expertise regarding cancer-related cognitive problems at work since  Need for a screening program for long-term effects of cancer, including cancer-related cognitive problems to facilitate professionals to offer adequate care for cancer survivors who deal with cancer-related cognitive problems at work. |
| 16. | Knott et al. (2014) | Participants were unclear about who would conduct this assessment and identified a range of professionals; including cancer care coordinators, medical oncologists, general practitioners and occupational therapists. OHPs spoke frequently about the necessity of a multidisciplinary approach. | Need for a multidisciplinary approach in the RTW of cancer survivors, including e.g. cancer care coordinators, medical oncologists, general practitioners and occupational therapists. |
| 17. | Lamort-Bouche et al. (2020) | BC specialists reported a lack of opportunity to discuss work-related issues of their patients. Work and RTW issues were never on the agenda of the weekly multidisciplinary meeting, which was exclusively dedicated to treatment issues | Need for opportunities to discuss work-related issues of their patients |
|  |  | Lack of time, lack of training and sometimes lack of interest were mentioned about patients' work-related issues. Several respondents expressed their inability to meet the work-related expectations of BC patients. | Need for time, training and interest about patients’ work-related issues |
| 18. | Leensen et al. (2018) | However, all care providers indicated a need for more mutual consultation, in person. | Need for more mutual consultation with other health care professionals. |
| 19. | MacLennan et al. (2017) | Collectively, participants recognised that there is a need for a better system for providing work-related information, advice and related support to people with urological cancer. | Need for a better system for providing work-related information, advice and related support to people with urological cancer Need for training on communication skills |
|  |  | Many demonstrated a lack of awareness of possible information, advice and related support resources. | Need for awareness of advice and related support resources. |
|  |  | Many participants believed that training should involve improving stakeholders’ communication skills in relation to both their competence and confidence. | Need for training on communication skills. |
| 20. | Marinas-Sanz et al. (2023) | NR | NR |
| 21. | Martin et al. (2022) | It is important to note that a lack of resources was outlined in each center, although each center included a social service in which patients could theoretically receive support for their RTW. However, as pointed out by social workers, there is a lack of resources to address the concerns of all patients; thus, only the most vulnerable patients can benefit from their support, and RTW is only one of several concerns that social workers address with these patients | Need for resources to support all cancer survivors with their RTW |
| 22. | Olischlager et al. (2023) | NR | NR |
| 25. | Rondinone et al. (2023) | NR | NR |
| 26. | Sarfo et al. (2023) | Some GPs explained that it can be difficult to get in contact with OPs when needed. | Need to get in contact with OPs when needed easily. |
|  |  | One GP mentioned that he is not willing to contact the OP anymore, rather the OP should initiate contact. | Need that OPs should initiate contact |
|  |  | To provide work guidance, GPs believe they need to be aligned with OPs, or at least have the opportunity to discuss with OPs. | Need to aligned with OPs, or at least have the opportunity to discuss with OPs. |
|  |  | OPs described that there is a shared goal between GPs and OPs in improving patients’ return to work. As such, they mentioned that they expect GPs to be open to a discussion with them about work-related issues of patients. However, OPs described sensing distrust among GPs regarding the role of OPs. They said that it may be helpful to better inform GPs about their function as they feel that this distrust may not be justified. | Need to inform GP’s about role of OP’s, because OP’s are sensing distrust among GP’s |
| 27. | Sheppard et al. (2023) | NR | NR |
| 28. | Shim et al. (2019) | NR | NR |
| 29. | Stergiou-Kita et al. (2016)^1^ | NR | NR |
| 30. | Stergiou-Kita et al. (2016)^2^ | P6: I don’t have the luxury of seeing them do their job which would be much more objective. So I just ask them what they do, ask them what they think they can do, what they think they can’t do. So it’s really just their perceptions that I’m getting. I’m not getting an accurate, objective assessment. | Need to objectively assess functional work abilities |
|  |  | Recommendations regarding work readiness could also be hampered by the lack of available resources (such as funding) to complete more in-depth functional abilities evaluations, as discussed by the following vocational service provider  P3: In some cases I encourage doctors to ask for assessments, physical, functional capacity evaluations. But right now in our health care system it’s not provided. We don’t have that access [to functional capacity evaluations], [unless] it has to be asked for from a third party insurance provider. So I encourage physicians to say I am not going to clear this person [to return to work]…I think he needs to get this [additional] opinion [functional evaluation]. But sadly that’s not available for someone who doesn’t have LTD [long-term disability coverage]. | Need for available resources (such as funding) to complete more in-depth functional abilities evaluations |
|  |  | Third, were expressed concerns about poor support for communication and collaborations between health care providers and community vocational support programs that could fill the current gaps in work-related supports. | Need for communication and collaborations between health care providers and community vocational support programs that could fill the current gaps in work-related supports |
| 31. | Tamminga et al. (2016) | NR | NR |
| 35. | Zaman et al. (2017) | Lack of financial support in the form of a salary for the OOP, since work is still voluntarily | Need for financial support in the form of a salary |
| 36. | Zaman et al. (2020)^3^ | In response to the open questions, however, two said that they [nurses] would have liked a refresher course. | Need to have a refresher course |
|  |  | The OOPs were unanimous that the time available for the first meeting was too short to provide this kind of support. | Need to have more time for the first consultation |
| 37. | Zaman et al. (2020)^4^ | Another major barrier for the oncological occupational physicians is financial support. This kind of care is not yet covered by standard Dutch medical insurance, which basically means that the oncological occupational physicians have to provide it on a voluntary basis. | Need for financial support for providing their support to patients |
|  |  | One oncological occupational physician mentioned a need for easily accessible collegial consultations, which were not feasible during this study for logistical reasons. | Need for easily accessible collegial consultations |
|  |  | Suggestions for improvement from the healthcare professionals included bridging the gap between hospital treatment and RTW, enabling the oncological occupational physician to translate information from primary care concerning work-related problems to help in the patient’s transition to that of their regular occupational physician (although this would require the patient’s consent) | Need to bridge the gap between hospital treatment and RTW enabling the oncological occupational physician to translate information from primary care concerning work-related problems to help in the patient’s transition to that of their regular occupational physician |
|  |  | and making the oncological occupational physician part of the multidisciplinary treatment program within the clinical setting | Need to make oncological occupational physician part of the multidisciplinary treatment program |
| 38. | Zaman et al. (2018) | A mentioned missing element by the experts was the connection between clinical setting and work environment. | Need to have a connection be-tween clinical setting and work environment |
|  |  | The omission of the relationship with the employer and the regular occupational physician of the patient was also mentioned | Need to have a relationship with the employer and the regular occupational physician of the patient |
| 39. | Zegers et al. (2023) | NR | NR |
| 40. | Zegers et al. (2022) | Several participants indicated that in order to optimize the work participation process of patients with advanced cancer, a stakeholder network should be established around the patient. Currently, some vital linking pins were said to be missing. | Need for a stakeholder network around the patient. |
|  |  | According to participants, work should first be discussed within the general health care system, for example, at the hospital or at the general practitioner's (GP's) office. | Need to discuss work within the general health care system |

^1^ = Stergiou-Kita et al. et al. (2016): The provision of workplace accommodations following cancer: survivor, provider, and employer perspectives

^2^ = Stergiou-Kita et al. (2016): Am I ready to return to work? Assisting cancer survivors to determine work readiness

^3^ = Zaman et al. (2020): Process evaluation of a tailored work-related support intervention for patients diagnosed with gastrointestinal cancer

^4^ = Zaman et al. (2020): Is a tailored work-related support intervention feasible in everyday clinical practice? The experience of healthcare professionals and patients with cancer

NR = Not reported

**Table 4b. Identified support needs of employers**

| **#** | **Authors (year)** | **Extracted from full text** | **Support need** |
| --- | --- | --- | --- |
| 1. | Algeo et al. (2022) | NR | NR |
| 3. | Bilodeau et al. (2024) | NR | NR |
| 4. | Boelhouwer et al. (2021) | NR | NR |
| 6. | De Boer et al. (2017) | ondersteuning bij regelen geschikte en gewenste werkaanpassingen | Need for support to arrange wanted and suitable work adaptations. |
|  |  | richtlijnen bij re-integratie. | Need for return to work guidelines |
|  |  | HR adviseurs en leidinggevenden willen meer ondersteuning van de bedrijfsarts of van een externe deskundige voor henzelf en voor de werknemer. | Need for more support from the occupational physician or other external expert |
| 7. | De Rijk et al. (2020) | Firstly, employers express the need for structured procedures. | Need for structured procedures; |
|  |  | Secondly, many employers emphasize collaboration with other stakeholders, among which the role of the occupational physician or occupational health specialist is highly appreciated. | need to collaborate with other stakeholders; |
|  |  | Thirdly, most employers refer to communication skills training. | need for communication skills training |
|  |  | Fifthly, employers refer to the need for financial resources for them to assist workers with cancer. | need for financial resources to assist workers with cancer |
|  |  | The goals of this problem solving includes staying in touch with the worker in order to ensure that a worker’s needs are clear and to collect necessary information on the course of the illness and the treatment via the worker. | Need to stay in touch with worker to ensure worker's needs are clear and to collect necessary information on the course of illness and treatment. |
| 10. | Fassier et al. (2018) | At the time of the first RTW, all the workplace respondents agreed on the lack of preparation between actors [breast cancer survivor (BCS), physicians, and employer], while coordination was deemed necessary. | Need for coordination between actors (arts, wherever, patient) at the time of the first RTW |
|  |  | It was emphasized that both colleagues and management needed information and training to support a woman returning to work after cancer. | Need for Information and training to support a woman returning to work after cancer |
|  |  | and the management (dealing with confidentiality). | Need of support in dealing with confidentiality. |
| 11. | Fitch and Nicoll (2019) | Participants generally agreed that a better understanding of the employee's situation, stronger communication between key stakeholders (medical team and insurers), increased awareness of options and strategies available, and a return to work after cancer toolkit would increase the likelihood of successfully managing such workplace situations. | Need for a return to work after cancer toolkit |
|  |  | A key issue for employees and employers is adequate financial support. Employee benefits vary widely, largely in relation to the size of the employer. Financial pressure can be a significant factor in effectively managing an employee's position during their illness and return to work. Participants, particularly those in small and midsized organizations, had little awareness of programs and services available outside their organizations for people dealing with cancer. | Need for financial support |
| 12. | Greidanus et al. (2021) | Participants were motivated to use the intervention by intrinsic as well as extrinsic factors. As to the intrinsic facilitators, participants indicated that they felt responsible and were motivated to provide the best possible support to the cancer survivor:  I’m just very intrinsically motivated to support colleagues, in any form whatsoever (…) and if you are diagnosed with cancer (…) that simply does a lot with somebody. I really want as many tools as possible (…) to get someone through that | Need for support on how to provide the best possible support to cancer survivors, tailored to the specific situation of their cancer survivor (e.g. in terms of RTW phase) |
|  |  | Lastly, participants suggested that the intervention should include the possibility to engage a specialized coach for situations that are more complex | Need for support by a specialized service for situations that are more complicated of complex |
|  |  | Nevertheless, the phases were not adequate for all situations, and the participants suggested adding a 5th phase to the intervention:  …the question is whether [this cancer survivor] will survive. So, those are really tough things. Then it does not make sense to say, well, how are we going to plan your return to work. But you are still the manager and it is your task to, let’s say, guide this in a different way, and I miss that a little in the [intervention]. | Need for support on how to provide support to cancer survivors who will not be able to return to work and/or will not survive. |
|  |  | Some participants indicated that they missed a particular type of cancer survivor, for example a non-cooperative cancer survivor who wants to slow down the RTW or an even more emotional cancer survivor. | Need for support on how to support non-cooperative cancer survivors who want to slow down the RTW, or more emotional cancer survivor. |
| 13. | Grus et al. (2019) | Need for institutional flexibility to provide individualized accommodations for the cancer survivor (limitations in existing organizational policies and practixes that formally or informally guided the treatment of employees with illnesses). | Need for institutional flexibility to provide individualized accommodations for the cancer survivor |
|  |  | Need for open communication with the cancer survivor early in their disease experience to ensure that the cancer survivor have timely access to all available legal and organizational services. | Need for open communication with the cancer survivor early in their disease experience to ensure that the cancer survivor have timely access to all available legal and organizational services. |
|  |  | Need for better communication with frontline managers (who serve as the cancer survivors’ primary point of contact) to inform them about leave availability for cancer survivors. | Need for better communication with frontline managers (who serve as the cancer survivors’ primary point of contact) to inform them about leave availability for cancer survivors. |
|  |  | Clear processed (e.g. up-to-date organizational policies) | Need for a clear process ( (e.g. up-to-date organizational policies) |
|  |  | and education to management teams, supervisors and managers. | Need for education to management teams, supervisors and managers. |
|  |  | Developing ongoing, standardised communication between human resource administrators and frontline managers about their employees' disease‐related needs and changes. | Need for ongoing, standardised communication between human resource administrators and frontline managers about their employees' disease‐related needs and changes. |
| 19. | MacLennan et al. (2017) | NR | NR |
| 20. | Marinas-Sanz et al. (2023) | Moreover, they pointed out the necessary collaboration between the health professionals who follow the patient and the specialists in Occupational Medicine in the company | Need for collaboration between the health professionals who follow the patient and the specialists in Occupational Medicine in the company |
| 23. | Petersen et al. (2019) | NR | NR |
| 24. | Popa et al. (2020) | On the negative side, employers emphasized that the lack of legislation for supporting the employee after returning, mostly the legislation regarding vocational rehabilitation, was a major barrier against adjusting to work | Need for legislation for supporting the employee after returning, mostly the legislation regarding vocational rehabilitation, was a major barrier against adjusting to work |
|  |  | The lack of collaboration with specialists (health professionals, psychologist, social worker) was another factor hindering a proper readjustment at work. | Need for collaboration with specialists (health professionals, psychologist, social worker) |
| 29. | Stergiou-Kita et al. (2016)^1^ | NR | NR |
| 31. | Tamminga et al. (2016) | NR | NR |
| 32. | Tiedtke et al. (2020) | NR | NR |
| 33. | Tiedtke et al. (2017) | NR | NR |
| 34. | Tiedtke et al. (2014) | Some of them felt there should have been a RTW policy within the organization or guidelines | Need for a RTW policy |
|  |  | in this respect they felt isolated because there were few opportunities to discuss it with other fellow employers or doctors. | Need to discuss with fellow employers or doctors |

^1^ = Stergiou-Kita et al. et al. (2016): The provision of workplace accommodations following cancer: survivor, provider, and employer perspectives

NR = Not reported

***Article title***

A scoping review on information and support needs regarding Cancer and Work: Part II – needs of employers and professionals in supporting cancer survivors

***Journal***

Journal of Cancer Survivorship

***Authors***

J.M. Mollet^1,2^, M.A. Greidanus^1,2^, S.F.A. Duijts^3,4,5^, A.G.E.M. de Boer ^1,2,5^, P.M.V Janse^1,2^, I.L. Bakker^1,2^, S.J. Tamminga^1,2^

***Affiliation***

1: Amsterdam UMC location University of Amsterdam, Department of Public and Occupational Health, Amsterdam, The Netherlands

2: Amsterdam Public Health research institute, Societal Participation and Health, Amsterdam, The Netherlands

3: Netherlands Comprehensive Cancer Organization (IKNL), Research and Development, Utrecht, Netherlands

4: Amsterdam UMC location Vrije Universiteit, Department of Medical Psychology, Amsterdam, The Netherlands

5: Cancer Center Amsterdam, Cancer Treatment and Quality of Life, Amsterdam, The Netherlands

***Corresponding author***

Name: Judith Mollet

E-mail address: j.m.mollet@amsterdamumc.nl
